# Biomimetic-Nanoparticle-Enhanced Photothermal Immunotherapy: Targeted Delivery of Near-Infrared Region II Agents and Immunoadjuvants for Tumor Immunogenicity

**DOI:** 10.34133/bmr.0151

**Published:** 2025-03-04

**Authors:** Yanlu Yu, Wen Li, Qiqi Yu, Jingtao Ye, Hu Wang, Yang Li, Shouchun Yin

**Affiliations:** ^1^ Key Laboratory of Organosilicon Chemistry and Materials Technology of Ministry of Education, College of Materials, Chemistry and Chemical Engineering, Hangzhou Normal University, 311121 Hangzhou, P. R. China.; ^2^ Key Laboratory of Ageing and Cancer Biology of Zhejiang Province, Institute of Ageing Research, School of Medicine, Hangzhou Normal University, 311121 Hangzhou, P. R. China.

## Abstract

Advancing at the cutting edge of oncology, the synergistic application of photothermal therapy coupled with immunotherapy is rapidly establishing itself as an innovative and potent strategy against cancer. A critical challenge in this domain is the precise and efficient targeting of tumor tissues with photothermal agents and immunoadjuvants while minimizing interference with healthy tissues. In this paper, we introduce an ingenious biomimetic nanoparticle platform, cancer cell membrane coated F127/(R837 and IR1048) (CFRI) nanoparticles encapsulating a near-infrared region II photothermal agent, IR1048, and an immunostimulatory molecule, R837, with their surface modified using membranes derived from tumor cells, conferring exceptional specificity for tumor targeting. CFRI nanoparticles demonstrated an extraordinary photothermal conversion efficiency of 49%, adeptly eradicating in situ tumors. This process also triggered the release of damage-associated molecular patterns, thereby activating dendritic cells and catalyzing the maturation and differentiation of T cells, initiating a robust immune response. In vivo animal models substantiated that the CFRI-mediated synergistic photothermal and immunotherapeutic strategy markedly suppressed the proliferation of in situ tumors and provoked a vigorous systemic immune response, effectively curtailing the metastasis and recurrence of distant tumors. The successful development of the CFRI nanoparticle system offers a promising horizon for future clinical translations and pioneering research in oncology.

## Introduction

Photothermal therapy (PTT) has ascended as a vanguard in cancer treatment, distinguished by its noninvasive profile, minimal medication dosage, minimally invasive procedures, and diminished potential for drug resistance [1,2]. PTT operates through the conversion of external near-infrared (NIR) light into thermal energy by photothermal agents (PTAs), a process that induces thermal damage and apoptosis in tumor tissues [3–7]. Gold nanoparticles, as exemplars of PTAs, have illustrated remarkable efficacy in the thermal ablation of tumor tissues across preclinical models [8–13]. Nonetheless, PTT encounters challenges in tackling tumor metastasis and recurrence, spurring the quest for synergistic therapeutic approaches [14–16]. Immunotherapy, mediated by cytotoxic T lymphocytes (CTLs), targets tumor-specific antigens to neutralize tumor cells, offering important promise against metastasized or potentially metastatic tumor cells [17–19]. Emerging research has highlighted that PTT can precipitate immunogenic cell death (ICD) during the ablation of primary tumors, leading to the extracellular release of intracellular molecules such as adenosine triphosphate (ATP), high mobility group box 1 (HMGB1), and calreticulin (CRT), collectively classified as damage-associated molecular patterns (DAMPs) [20–29]. These DAMPs are identifiable by Toll-like receptor agonists, triggering an immune response [30]. The utilization of Toll-like receptor agonists, such as R837, as immunological adjuvants can augment the recognition of DAMPs generated by PTT, fostering the differentiation and maturation of immature dendritic cells (DCs) upon signal reception and fortifying their capacity to eradicate primary and metastatic tumor cells [31]. However, achieving synergistic therapeutic efficacy hinges on the effective and secure delivery of PTAs and immunological adjuvants to the tumor tissues while minimizing impact on healthy tissues [32,33].

The advent of nanomedical science has catalyzed innovative resolutions to these challenges [34–36]. Nanoparticles, as vectors for drug delivery, enhance drug solubility, stability, and bioavailability while curtailing toxicity to normal tissues [37–39]. Biomimetic nanoparticles, in particular, emulate the structure and function of biological molecules or cells, enabling precise targeting and potent treatment of tumors [40–43]. Cell membranes from red blood cells, immune cells, and cancer cells, which are pivotal in intercellular communication and immune defense, are endogenous substances within the biological system [44–48]. The transference of these cell membranes onto nanoparticles allows for stealth and camouflage, circumventing the body’s immune clearance mechanisms, thus achieving immune evasion and prolonging circulation time and lifespan [49,50]. Moreover, these nanoparticles retain the natural physicochemical properties of the membranes, inheriting their unique biological functions, enhancing cellular endocytosis, and employing antigen-specific recognition on the cell surface for active targeting of tumor cells, with homologous targeting by the same type of cancer cell membrane being exceptionally effective.

**Fig. 1.**
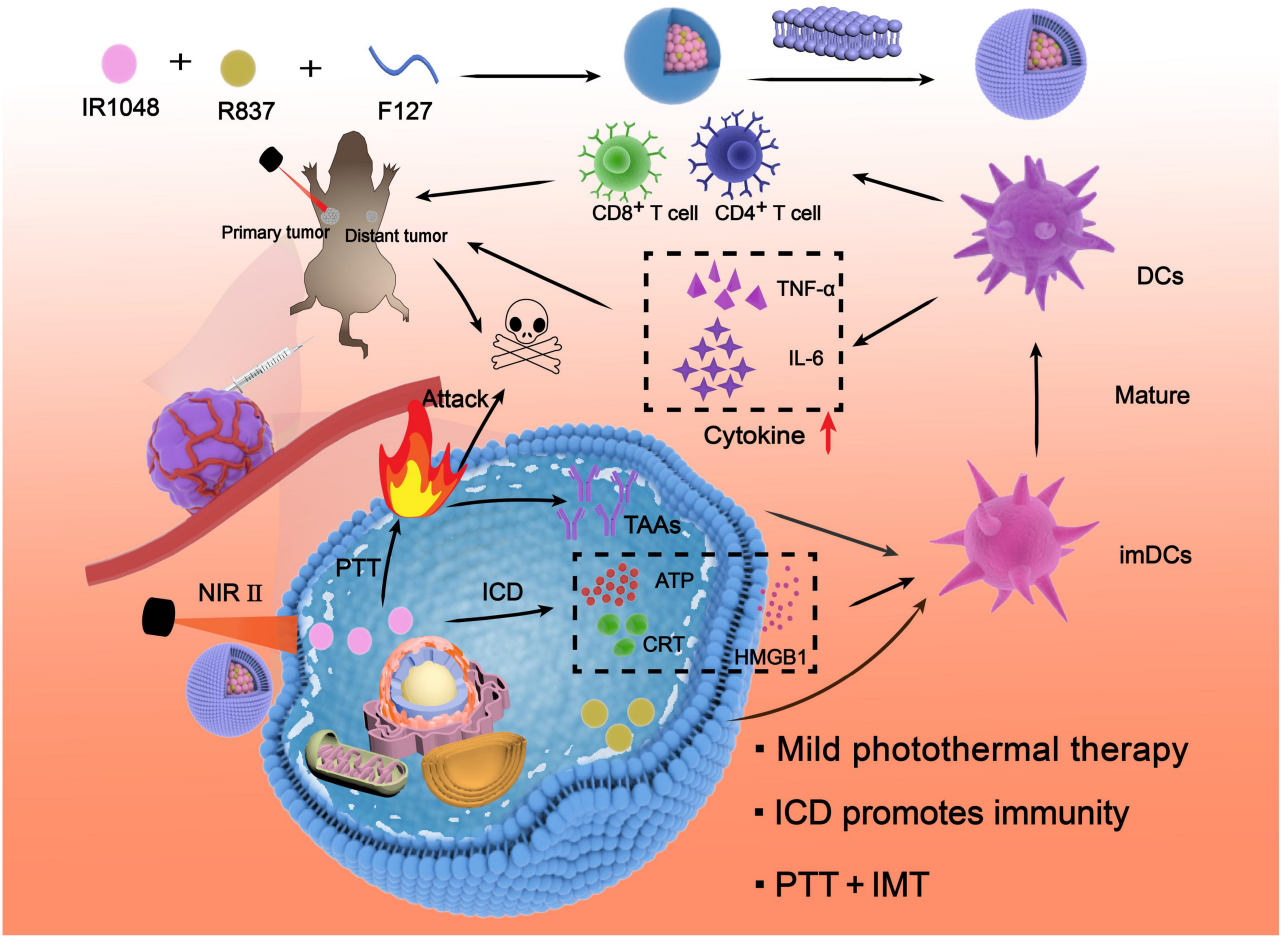
Schematic diagram of design and mechanism of action of biomimetic nanoparticles (NPs) (cancer cell membrane coated F127/(R837 and IR1048) [CFRI]) loaded with the photothermal agent IR1048 and the immune adjuvant R837. DCs, dendritic cells; TNF-α, tumor necrosis factor-α; IL-6, interleukin-6; imDCs, immature dendritic cells; NIR-II, near-infrared region II; PTT, photothermal therapy; ICD, immunogenic cell death; TAAs, tumor-associated antigens; ATP, adenosine triphosphate; CRT, calreticulin; HMGB1, high mobility group box 1; IMT, immunotherapy.

Herein, we have engineered a biomimetic nanodelivery system predicated on cancer cell membranes. By encapsulating the near-infrared region II (NIR-II) PTA IR1048 and the Toll-like receptor 7 (TLR7) agonist immunological adjuvant R837 within the amphiphilic polymer F127, we have synthesized amphiphilic nanoparticles and coated their surface with cancer cell membranes (4T1) to fabricate biomimetic nanoparticles (cancer cell membrane coated F127/(R837 and IR1048) [CFRI]). Throughout the therapeutic process, these biomimetic nanoparticles, veiled with cancer cell membranes, exploit the tumor’s enhanced permeability and retention effect and homologous targeting to successfully internalize into tumor cells (Fig. 1). They then gradually release the NIR-II PTA IR1048 and the immunological adjuvant R837 under tumor-specific acidic conditions. The PTA, upon light absorption, elevates the temperature, inflicting damage to and causing the demise of tumor cells while also facilitating the release of intracellular DAMPs such as CRT, HMGB1, and ATP into the extracellular environment through the process of ICD. The immunological adjuvant R837, functioning as a TLR7 receptor agonist, engages these DAMPs, upregulating major histocompatibility complex (MHC) molecules on DCs and prompting their maturation. This advancement enhances the presentation of tumor-associated antigens to naive T cells, driving their differentiation into regulatory T cells and cytotoxic T cells. These immune cells subsequently secrete cytokines to neutralize metastatic tumor cells, further amplifying the antitumor efficacy. Through rigorous evaluation via in vitro experiments and in vivo animal models, CFRI nanoparticles have demonstrated superior photothermal conversion efficiency and biocompatibility under 1,064-nm laser irradiation, effectively suppressing tumor growth both ex vivo and in vivo while also inciting a robust immune response. This work not only introduces an effective new strategy and material option for synergistic photothermal immunotherapy of cancer but also provides important experimental evidence for a deeper understanding of the ICD mechanism induced by PTT and the enhancement of tumor immunogenicity through nanoparticle engineering.

## Materials and Methods

### Materials

IR1048 (1-butyl-2-[2-[3-[(1-butyl-6-chlorobenz[*cd*]indol-2(1*H*)-ylidene)ethylidene]-2-chloro-1-cyclohexen-1-yl]ethenyl]-6-chlorobenz[*cd*]indol) and R837 (1-(2-methylpropyl)-4amino-1*H*-imidazolo[4,5-*c*]quinoline) were procured from GMA-Aldrich Trading Co. Ltd. 3-(4,5-Dimethylthiazol-2-yl)-2,5-diphenyltetrazolium bromide (MTT) and trypsin–EDTA were sourced from Biomics Biotechnologies Co. Ltd. (Nantong, China). A live/dead cell double-staining kit and an annexin V–fluorescein isothiocyanate (FITC)/propidium iodide (PI) double-staining apoptosis detection kit were acquired from KeyGEN BioTECH Co. Ltd. (Jiangsu, China). Fetal bovine serum (FBS) was supplied by Sijiqing (Hangzhou, China), and Dulbecco’s modified Eagle medium (DMEM) was obtained from Sigma-Aldrich (Missouri, USA). An ATP assay kit and Membrane and Cytosol Protein Extraction Kit were purchased from Beyotime (Shanghai, China). Enzyme-linked immunosorbent assay (ELISA) kits for the detection of tumor necrosis factor-α (TNF-α), interleukin-10 (IL-10), and interleukin-6 (IL-6), as well as antibodies, including anti-mouse F4/80, anti-CD206, anti-MHC II, anti-CD8, anti-CD3, anti-CD4, anti-CD62L, anti-CD44, anti-CD80, anti-CD86, and anti-CD11c, were all obtained from Dakewei Biotechnology (Shenzhen, China). Secondary antibodies, Alexa Fluor 488-conjugated goat anti-rabbit immunoglobulin G, anti-HMGB1, and anti-CRT, were purchased from Boster Biological Technology Co. Ltd. (California, USA).

### DLS and TEM analysis of materials

Transmission electron microscopy (TEM) images were obtained using a Hitachi S-4800 instrument. Dynamic light scattering (DLS) and zeta potential determinations were performed with a Malvern Zetasizer Nano ZS90. Absorption spectra were recorded on a Hitachi U-5300 spectrophotometer. Flow cytometry analyses were conducted using a CytoFLEX S system. Confocal fluorescence imaging of cells was accomplished with an LSM800 laser scanning microscope (Zeiss). Thermal infrared (IR) images were captured by a FLIR ONE Pro thermal camera.

### Preparation and characteristics of nanoparticles

IR1048 (1.5 mg) was dissolved in a dimethyl sulfoxide (DMSO) solution (1.5 ml), and R837 (1.5 mg) was dissolved in an acetic acid solution (1.5 ml). Subsequently, F127 (30 mg) was dispersed in 30 ml of deionized water. The combined solution of IR1048 and R837 was added dropwise to the F127 aqueous solution under ultrasonication at a power of 35 W, facilitating the formation of self-assembled micelle dispersions. The resulting nanoparticle solution was then transferred into a dialysis bag with a molecular weight cutoff of 4,000 to 6,000 Da and dialyzed against deionized water for 12 h to eliminate any unencapsulated drugs, salts, and residual solvents such as DMSO and acetic acid. The resulting FRI nanoparticles were freeze-dried and subsequently weighed for subsequent use.

### Encapsulation of cancer cell membranes

To isolate the 4T1 cancer cell membrane, 4T1 cells were harvested via centrifugation and resuspended in hypotonic lysis buffer supplemented with a protease inhibitor. The cells were incubated on ice for 12 min and then subjected to 3 cycles of freezing–thawing in liquid nitrogen and 37 °C water bath. The mixture was then centrifuged at 700 *g* for 10 min at 4 °C, and the supernatant was carefully collected. This supernatant was further centrifuged at 14,000 *g* for 30 min at 4 °C, and the resulting pellet, enriched with the 4T1 cell membrane, was collected. The isolated membrane was subsequently mixed with FRI and extruded through a 400-nm-pore-sized polycarbonate membrane at least 5 times to produce CFRI.

The nanoparticles were characterized for absorbance at 1,100 and 200 nm using an ultraviolet (UV)–visible–NIR spectrophotometer. The drug encapsulation efficiency and drug loading efficiency were calculated using the following formulas:

$$\begin{array}{c} \text{Drug encapsulation efficiency}\;\left(\%\right)=\\ \left(\text{drug mass in CFRI}\right)/\left(\text{initial drug mass}\right)\times 100\% \end{array}$$ (1)

$$\begin{array}{c} \text{Drug loading efficiency}\;\left(\%\right)=\\ \left(\text{drug mass in CFRI}\right)/\left(\text{total mass of CFRI}\right)\times 100\% \end{array}$$ (2)

### DLS and TEM analysis of CFRI

The hydrodynamic diameter and surface change of CFRI were characterized using DLS. For TEM analysis, the nanoparticle aqueous solution was applied dropwise onto a 200-mesh carbon-film-coated copper grip, followed by the addition of phosphotungstic acid solution for negative staining.

### Cell membrane proteins analysis

The cancer cell membrane proteins associated with CFRI were analyzed using sodium dodecyl sulfate polyacrylamide gel electrophoresis (SDS-PAGE). Protein concentrations in various samples were determined using the bicinchoninic acid assay kit. To prepare the samples, protein loading buffer was added to each sample at a volume ratio of 1:4 (*v*/*v*), followed by heating in a metal bath at 100 °C for 5 min to ensure complete protein denaturation.

Equal amounts of protein (10 μg/well) were loaded onto a 10% SDS-PAGE gel, and electrophoresis was performed using SDS-PAGE Gel Rapid Prep Kit according to the manufacturer’s protocol. The separated proteins were visualized by staining with Coomassie blue and then documented after destaining in water for 12 h.

### Photothermal performance in vitro

Free IR1048, FRI, and CFRI, each at a constant concentration of IR1048 (2 μg/ml), were introduced into separate 1.5-ml tubes. The samples were then subjected to NIR laser irradiation (1,064 nm, 1.0 W/cm^2^) for 10 min, with IR imaging captured at 1-min intervals.

### Concentration-dependent photothermal property

Aqueous solutions of CFRI at varying concentrations (1, 2, 5, 10, and 20 μg/ml) were subjected to 1,064-nm laser irradiation at a power of 1.0 W/cm^2^ for 10 min. Temperature changes were monitored in real time using an FLIR thermal imaging camera.

### Power-density-dependent property

The CFRI aqueous solution, prepared at a concentration of 2 μg/ml, was exposed to 1,064-nm laser irradiation at varying power densities (0.5, 0.8, 1.0, and 1.5 W/cm^2^) for 10 min. Temperature changes were captured using an FLIR thermal camera.

### Photothermal stability

The CFRI aqueous solution (2 μg/ml) was subjected to laser irradiation (1,064 nm, 1.0 W/cm^2^) for 10 min, followed by cooling down to the initial temperature upon cessation of the laser. This temperature cycling, monitored by a FLIR thermal camera, was repeated for a total of 5 cycles.

### Cell culture and cell uptake

4T1 cells were maintained in DMEM supplemented with 10% FBS, 1% penicillin (50.0 IU ml^−1^), and streptomycin (50.0 IU ml^−1^) under conditions of 37 °C, 5% CO_2_, and saturated humidity. For the cellular uptake study, 4T1 cells were plated in 8-well chambered coverslips at a seeding density of 1.5 × 10^4^ cells/well and incubated for 24 h. The culture medium was subsequently replaced with DMEM containing free IR1048, FIR1048, FRI, or CFRI, all at a uniform concentration of 2 μg/ml of IR1048. Following a 4-h incubation, cells were rinsed with phosphate-buffered saline (PBS) to remove the test solutions and fixed in 4% (*w*/*v*) paraformaldehyde for 5 min at room temperature. Cells were then stained with 4′,6-diamidino-2-phenylindole (DAPI; to stain nuclei blue) for 10 min and rinsed twice with PBS, and fresh DMEM was added before visualization with confocal laser scanning microscopy (CLSM). The extent of cellular uptake was quantified using a flow cytometer.

### In vitro homologous targeting ability

To assess the targeted delivery capability of the 4T1 cancer cell membrane, a panel of cell lines including MCF-7 cells, U87 cells, B16 cells, HeLa cells, HepG2 cells, L929 cells, and 4T1 cells were cultured in 24-well plates at a density of 2 × 10^4^ cells/well for 24 h. The culture medium was then replaced with CFRI (at a concentration of 2 μg/ml in serum-free medium). After a 4-h incubation, CFRI was removed with PBS, and the cellular uptake of CFRI was quantified using flow cytometry across the different cell types.

### Cell uptake ability of different materials

The material-specific targeting efficacy of 4T1 cancer cell membranes was assessed. 4T1 cells were first cultured in 24-well plates at a density of 2 × 10^4^ cells/well for 24 h. The culture medium was then switched to DMEM containing either free IR1048, FIR1048, FRI, or CFRI, all at a uniform concentration of 2 μg/ml of IR1048. Following a 4-h incubation, the cells were rinsed with PBS to eliminate the varied treatment solutions, and flow cytometry was employed to measure the internalization of the different materials by 4T1 cells.

### In vitro cell viability

The cytotoxicity of CFRI was assessed through MTT assays. 4T1 cells were plated at a density of 1 × 10^6^ cells/well in a 96-well plate and cultured for 18 h. Subsequently, the culture medium was replaced with fresh medium containing varying concentrations of FIR1048, FR837, and CFRI (ranging from 0 to 5 μg/ml in terms of IR1048 concentrations). Following a 4-h incubation, the cells were exposed or not to a 1,064-nm NIR laser for 2 min at a power density of 1.0 W/cm^2^. The cells were then further incubated for an additional 18 h before the medium was replaced with an MTT solution (0.5 mg/ml in medium). After a 4-h incubation, the MTT solution was removed, and 100 μl of DMSO was added to each well to dissolve the formazan crystals for 15 min. Once the formazan crystals were fully dissolved, the optical absorbance (OD) at 570 nm was determined using a microplate reader.

### Live/dead cell staining assay

Live/dead staining was employed to investigate the in vitro antitumor effects of CFRI. 4T1 cells were seeded in a 24-well plate at a density of 2 × 10^4^ cells/well and incubated for 24 h. The culture medium was subsequently replaced with DMEM containing free IR1048, FIR1048, FRI, or CFRI, all at an IR1048 concentration of 2 μg/ml. The cells were then exposed to NIR irradiation (1,064 nm, 1.0 W/cm^2^, 2 min). Postirradiation, the cells were stained with calcein-AM/PI for 30 min and examined using a fluorescence microscope to visualize the viability and death of the cells.

### Cell apoptosis assay

Apoptosis assays were conducted to evaluate the in vitro antitumor efficacy of CFRI. 4T1 cells were inoculated into a 12-well plate and divided into different treatment groups. Following this, the cells were subjected to NIR irradiation (1,064 nm, 1.0 W/cm^2^, for 2 min). The treated cells were then stained with annexin V–FITC/PI for 30 min, rinsed with PBS, and harvested for analysis using a flow cytometer.

### Immunogenic induction cell death

The expression of CRT on 4T1 cells was assessed using immunofluorescence and flow cytometry. 4T1 cells were plated in 12-well plates at a density of 1 × 10^6^ cells/well and incubated for 24 h. The culture medium was then removed, and the cells were treated with DMEM, FR837, FIR1048, FRI, or CFRI, each at a uniform IR1048 concentration of 2 μg/ml, for 4 h. Subsequently, the irradiation groups were exposed to 1,064-nm NIR laser irradiation at a power of 1.0 W/cm^2^ for 2 min and further cultured for an additional 18 h. Posttreatment, the supernatant was removed, and the cells were incubated with anti-CRT antibody after washing 3 times with cold PBS. Following a 30-min incubation, the cells were washed and incubated with an Alexa 488-conjugated monoclonal secondary antibody for 30 min. The 4T1 cells were then harvested and analyzed using flow cytometry. Concurrently, the cells were fixed with 0.25% paraformaldehyde for 15 min and finally stained with DAPI before being visualized with CLSM.

The intracellular distribution of HMGB1 was evaluated using a similar immunofluorescence and flow cytometry approach. 4T1 cells were seeded in 12-well plates at a density of 1 × 10^6^ cells/well and incubated for 24 h. The medium was replaced with DMEM, FR837, FIR1048, FRI, or CFRI, each at a concentration of 2 μg/ml of IR1048, for 4 h. The irradiation groups were subjected to 1,064-nm NIR laser irradiation at a power of 1.0 W/cm^2^ for 2 min and cultured for an additional 18 h. Cells were fixed with 4% paraformaldehyde for 15 min and permeabilized with 0.1% Triton X-100 for 20 min. After a 45-min preincubation in PBS containing 1% FBS to block nonspecific binding, cells were incubated with an anti-HMGB1 antibody for 1 h, washed 3 times with cold PBS, and then incubated with an Alexa 488-conjugated secondary antibody for 45 min. The 4T1 cells were subsequently collected and analyzed using flow cytometry. Cells were also fixed with 4% paraformaldehyde for 15 min and stained with DAPI for visualization with CLSM.

Subsequently, the extracellular release of ATP was quantified using a commercial ATP assay kit. Briefly, 4T1 cells were plated in 12-well plates at a density of 1 × 10^6^ cells/well and incubated for 24 h. The culture medium was then aspirated, and the cells were treated with DMEM, FR837, FIR1048, FRI, or CFRI, each at a standardized IR1048 concentration of 2 μg/ml, for 4 h. Following this, the irradiation groups were subjected to 1,064-nm NIR laser irradiation at a power of 1.0 W/cm^2^ for 2 min and allowed to incubate for an additional 18 h. The supernatant from the cell cultures was then collected, and ATP levels were determined using an ATP assay kit according to the manufacturer’s protocol.

### In vitro DC maturation

The maturation of DCs was evaluated by flow cytometry. 4T1 cells were seeded in 12-well plates at a density of 1 × 10^6^ cells/well and incubated overnight to allow for cell adhesion. The cells were then treated with DMEM, FR837, FIR1048, FRI, or CFRI. Following a 4-h incubation, the irradiation groups were exposed to 1,064-nm NIR laser irradiation at a power of 1.0 W/cm^2^ for 2 min and further cultured for an additional 18 h. Subsequently, 1 × 10^6^ immature DCs were co-cultured with the pre-treated 4T1 cells for 24 h. The DCs were then stained with anti-CD11c–FITC, anti-CD86–phycoerythrin (PE), and anti-CD80–allophycocyanin (APC) antibodies, and their maturation was assessed by a flow cytometer.

### In vitro T cell assays

CTLs were quantified using flow cytometry. 4T1 cells were plated in 24-well plates at a density of 1 × 10^6^ cells/well and incubated overnight to facilitate cell attachment. The cells were then treated with DMEM, FR837, FIR1048, FRI, or CFRI. After a 4-h incubation, the irradiation groups were subjected to 1,064-nm NIR laser irradiation at a power of 1.0 W/cm^2^ for 2 min, followed by an additional 18 h of culture. Subsequently, the pre-treated 4T1 cells were coincubated with lymphocytes harvested from BALB/c mice for 24 h. The lymphocytes were labeled with anti-CD3–FITC, anti-CD4–peridinin–chlorophyll–protein (PerCP), and anti-CD8–PE antibodies, and the presence of CTLs was determined through flow cytometric analysis.

### In vitro cytokine detection

Cytokine secretion was assessed using ELISA. The concentrations of TNF-ɑ, IL-6, and IL-10 released from DCs following various treatments were determined using ELISA kits, according to the manufacturers’ standard protocols. Briefly, 4T1 cells cultured in 12-well plates were exposed to DMEM, FR837, FIR1048, FRI, or CFRI. After a 4-h incubation, the irradiation groups received 1,064-nm NIR laser irradiation at a power of 1.0 W/cm^2^ for 2 min. Following a 24-h incubation, the supernatant from these groups was collected and coincubated with DCs for an additional 24 h. The levels of secreted cytokines were then measured using ELISA kits, with the absorbance at 450 nm recorded using a microplate reader.

### Animals and tumor models

The mice were housed at the Laboratory Animal Center of Hangzhou Normal University with use license number SYXK (Zhejiang) 2020-0026 and cultivated in a pathogen-free environment with appropriate humidity and temperature. All animal experimental procedures were conducted in strict accordance with the protocols approved by the Institutional Animal Care and Use Committee and complied with the guidelines set forth by the State Department of Health. A bilateral 4T1 tumor-bearing mouse model was established by subcutaneously injecting 100 μl of a 4T1 cell suspension (1 × 10^6^ cells) into the right flank of female BALB/c mice to establish the primary tumor. Six days later, an identical number of cancer cells were injected subcutaneously into the left inguinal region to simulate a distant tumor (mimicking metastasis). Following 8 d of primary tumor growth, the mice were utilized for biodistribution analyses and therapeutic interventions.

### In vivo fluorescence imaging

BALB/c mice bearing 4T1 tumors were randomly assigned to 2 groups. Each group received a tail vein injection or orthotopic injection of 100 μl of PBS containing either FIR1048 or CFRI (with IR1048 at a concentration of 60 mg/ml). At predefined time points postinjection (1, 2, 4, 8, 12, 24, 48, 60, 72, and 120 h), the mice were imaged using the IVIS fluorescence imaging system. The fluorescence intensity in the primary tumors of each mouse was quantified using the Living Image software. At 120 h postinjection of the nanoparticles, the 4T1 tumor-bearing mice were humanely euthanized, and the primary tumors, hearts, livers, spleens, lungs, and kidneys were harvested and imaged using the IVIS fluorescence imaging system. The fluorescence intensity in the main organs and tumors was quantified using the Living Image software.

In vivo photoacoustic imaging of 4T1 tumor-bearing mice injected with FIR1048 (60 mg/ml, 100 μl) and CFRI (60 mg/ml, 100 μl) solutions were detected using multispectral optoacoustic tomography.

### In vivo tumor thermal imaging and temperature monitoring

4T1 tumor-bearing mice were randomly allocated to 3 groups. Each group received an intravenous injection of 100 μl of PBS solution containing either PBS, FIR1048, or CFRI (with IR1048 at a concentration of 60 μg/ml). Twenty-four hours postinjection, the primary tumor of each mouse was exposed to 1,064-nm laser irradiation at a power intensity of 1.0 W/cm^2^ for 10 min. Throughout the laser exposure, mice were visualized using an IR camera to monitor the temperature of the primary tumors.

### In vivo cancer therapy

BALB/c mice bearing 4T1 tumors were randomly assigned to 10 groups (*n* = 8/group). Eight days post-initial implantation, the mice were intravenously administered with either PBS, PBS + L, FR837, FR837 + L, FIR1048, FIR1048 + L, FRI, FRI + L, CFRI, and CFRI + L, with the concentration of IR1048 and R837 set at 2 mg/kg. Four hours after the injection, the tumors in the laser irradiation group were exposed to a 1,064-nm laser at a power density of 1.0 W/cm^2^ for 2 min. Subsequently, both the primary and distant tumor volumes, as well as the body weights of the mice, were monitored every 2 d over a period of 24 d. Tumor volume was calculated using the formula (tumor length) × (tumor width)^2^/2.

### Histological studies

On day 24, all groups of 4T1 tumor-bearing mice were humanely euthanized, and the tumors were harvested and fixed in 4% paraformaldehyde. The tissue samples were then embedded in paraffin, sectioned into 3-μm-thick slices, and subjected to hematoxylin and eosin (H&E) staining for microscopic examination to assess histological alterations. Apoptosis in tumor cells posttreatment was quantified using the terminal deoxynucleotidyl transferase-mediated dUTP nick-end labeling (TUNEL) method, while the proliferation rate of tumor cells was evaluated through Ki67 immunostaining, following the protocols provided by the manufacturers.

### In vivo biological safety experiment

Upon sacrifice of the tumor-bearing mice, the major organs (heart, liver, spleen, lung, and kidney) were carefully excised. The tissues were fixed in a 4% paraformaldehyde solution, embedded in paraffin, and sectioned into 3-μm-thick slices. The sections were then stained with H&E for histological examination. Concurrently, blood samples were collected from the eyeballs of the mice by enucleation before sacrifice, in preparation for subsequent biochemical and hematological analyses.

### In vivo examination of ICD

Sixteen days after the first administration, tumor tissues were extracted, frozen, and sliced. The slices were stained with anti-CRT or anti-HMGB1 primary antibodies overnight at 4 °C, followed by incubation with Alexa 488-conjugated secondary antibody and DAPI. The fluorescence signals of DAPI and Alexa 488 were observed by CLSM.

### In vivo evaluation of the T cell population

Upon completion of the treatment regimen, 4T1 tumor-bearing mice from each group were humanely euthanized. Tumors, spleens, and peripheral blood were harvested to isolate T cells. Spleen cells were first collected and treated with ACK Lysis Buffer to remove red blood cells. Subsequently, primary tumor tissues were excised, finely minced, and digested in PBS containing 1 mg/ml collagenase type IV at 37 °C for 4 h. The resulting cell mixture was filtered through a 200-mesh nylon sieve and treated with erythrocyte lysis buffer to prepare a single-cell suspension. Finally, the cell suspensions were incubated with anti-CD3–FITC, anti-CD4-PerCP, and anti-CD8–PE antibodies for 30 min and subsequently analyzed by flow cytometry.

### In vivo evaluation of DC maturation and macrophage phenotype

Upon completion of the treatments, 4T1 tumor-bearing mice from each experimental group were euthanized. The primary tumor tissues was minced into small fragments and enzymatically digested with 1 mg/ml collagenase type IV for 4 h at 37 °C. The cells were passed through a 200-mesh nylon filter and treated with erythrocyte lysis buffer to obtain a single-cell suspension. This suspension was aliquoted into 2 portions. One portion was stained with anti-CD11c–FITC, anti-CD86–PE, and anti-CD80–APC for 30 min to evaluate CD maturation using flow cytometry. The other portion was stained with anti-CD206–APC, anti-F4/80–PE, and anti-MHC II–BV421 to assess the phenotype of macrophages through flow cytometric analysis.

### In vivo determination of cytokine levels

Following the various treatments, serum was collected from each group of 4T1 tumor-bearing mice. The spleen lymphocytes, isolated as described earlier, were placed in a culture plate at a density of 20 million cells/plate and incubated for 24 h, and the supernatant was subsequently harvested by centrifugation. The concentrations of IL-6, IL-10, and TNF-α in serum, as well as those produced by the splenocytes, were quantified using ELISA kits according to the manufacturer’s protocol.

## Results

### Fabrication and characterization of CFRI nanoparticles

**Fig. 2.**
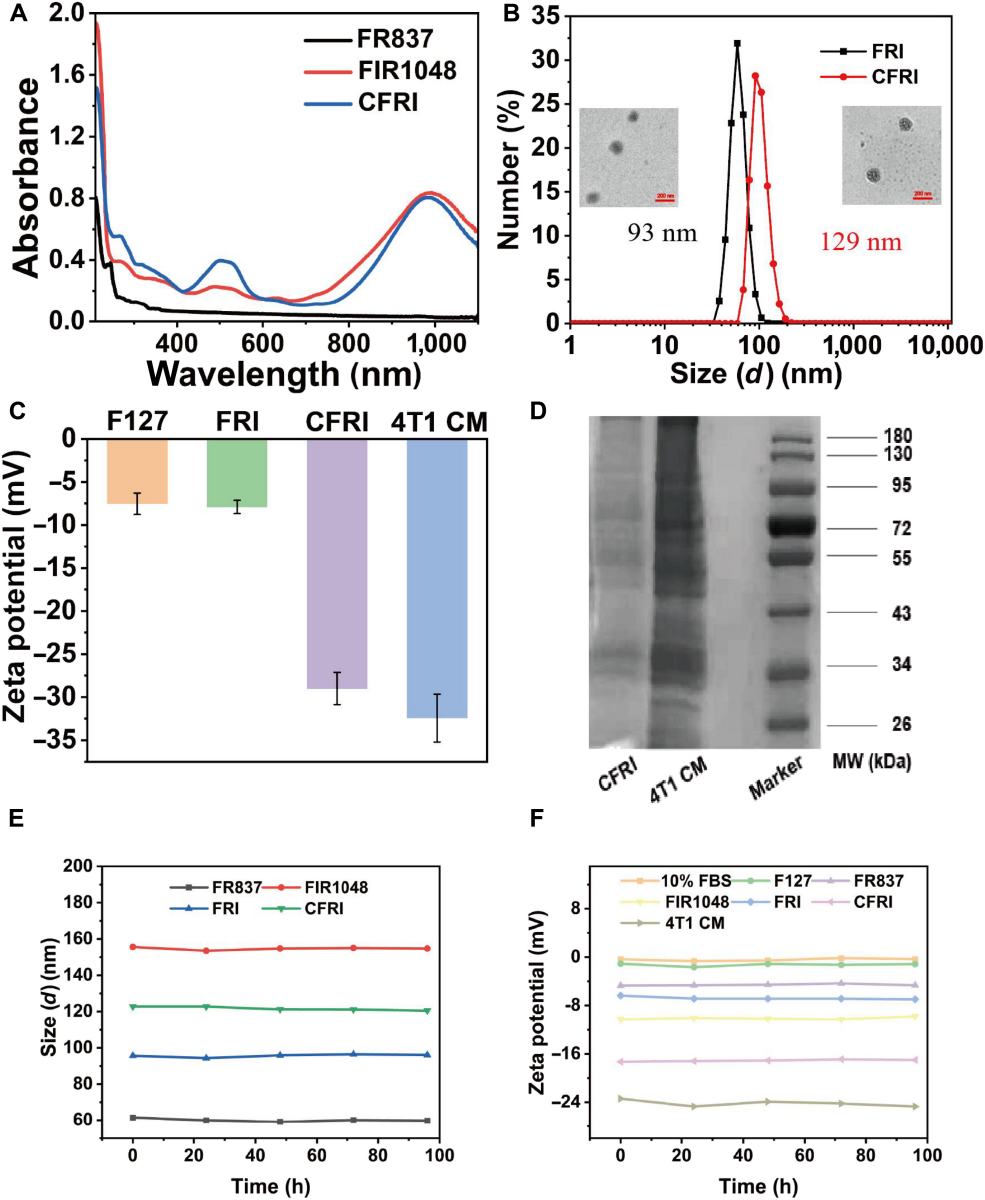
Characterization of CFRI nanoparticles. (A) Absorption spectra of FR837, FIR1048, and CFRI in water. (B) The size distribution of FRI nanoparticles and CFRI nanoparticles in water was determined by dynamic light scattering (DLS). (C) Zeta potentials of F127, FRI, CFRI, and 4T1 cell membrane (CM) in water. (D) Sodium dodecyl sulfate polyacrylamide gel electrophoresis (SDS-PAGE) protein analysis of 4T1 CM, FRI, and CFRI. (E) The particle size distributions of FR837 nanoparticles, FIR1048 nanoparticles, FRI nanoparticles, and CFRI nanoparticles in 10% fetal bovine serum (FBS) solution were determined by DLS within 96 h. (F) Zeta potentials of F127, FR837, FIR1048, FRI, CFRI, and 4T1 CM in 10% FBS solution. MW, molecular weight.

We have successfully synthesized an innovative class of biomimetic nanoparticles, termed CFRI nanoparticles, which encapsulates the synergistic therapeutic efficacy of photothermal and immunotherapy to achieve precise targeting and treatment of tumor tissues. The manufacturing process of CFRI nanoparticles entails the coencapsulation of the PTA IR1048 and the immunostimulatory agent R837 within an amphiphilic polymeric matrix of F127, with an exoskeleton derived from the 4T1 tumor cell membrane designed to enhance its targeting specificity within the tumor microenvironment. Employing UV–visible absorption spectroscopic analysis, the concurrent characteristic absorption peaks for IR1048 and R837 within the CFRI nanoparticles were observed, thereby confirming the coencapsulation of these therapeutic agents (Fig. 2A). Quantitative analysis elucidated an encapsulation efficiency of 1.98% for IR1048 and 1.56% for R837 (Fig. S1). DLS measurements revealed the marked impact of the cell membrane coating on nanoparticle dimensions, with the mean diameter of CFRI nanoparticles escalating from 93 nm for uncoated FRI to 129 nm (Fig. 2B). TEM imaging provided a visual representation of the morphology and architecture of both FRI and CFRI nanoparticles, which exhibited a regular spherical contour and a uniform size distribution. The surface coating on CFRI nanoparticles was particularly pronounced, establishing a robust structural foundation for their targeted interaction with tumor cells. Zeta potential measurements indicated a surface charge of −29.3 mV for CFRI nanoparticles, diverging from the −8.1 mV of uncoated FRI nanoparticles to closely approximate the −32.7 mV of the native 4T1 cell membrane, thereby corroborating the successful coating with the 4T1 cell membrane and endowing CFRI nanoparticles with analogous surface charge properties, which are pivotal for homologous targeting within tumor tissues (Fig. 2C). To substantiate the successful coating of the 4T1 cell membrane onto FRI nanoparticles, SDS-PAGE was implemented for an in-depth analysis of the protein composition on the CFRI nanoparticle surface. The protein band profiles on the CFRI nanoparticle surface were found to be highly congruent with those of the purified 4T1 cell membrane, confirming not only the successful coating but also the maintenance of membrane protein integrity, thus providing molecular-level evidence for the tumor-targeting capabilities of CFRI nanoparticles (Fig. 2D). To demonstrate the good stability of the materials under physiological conditions, the particle size changes and zeta potential changes of FIR1048 and FR837 nanoparticles in 10% FBS solution were continuously measured over a period of 5 d using DLS and zeta potential measurements (Fig. 2E and F). The results demonstrated that the zeta potential of FR837, FIR1048, FRI, CFRI, and 4T1 CM in 10% FBS solution was similar to that measured in pure water, with no potential change observed within the 5-d period. This indicates that the nanoparticles maintain their surface charge stability under physiological conditions, which is crucial for their interaction with biological systems. Furthermore, the particle sizes of FR837, FIR1048, FRI, and CFRI measured in 10% FBS solution were found to be stable and not different from those in pure water. This consistency in particle size suggests that the structural integrity of the nanoparticles is preserved in a physiological environment, which is essential for their in vivo applications.

### In vitro evaluation of photothermal performance

**Fig. 3.**
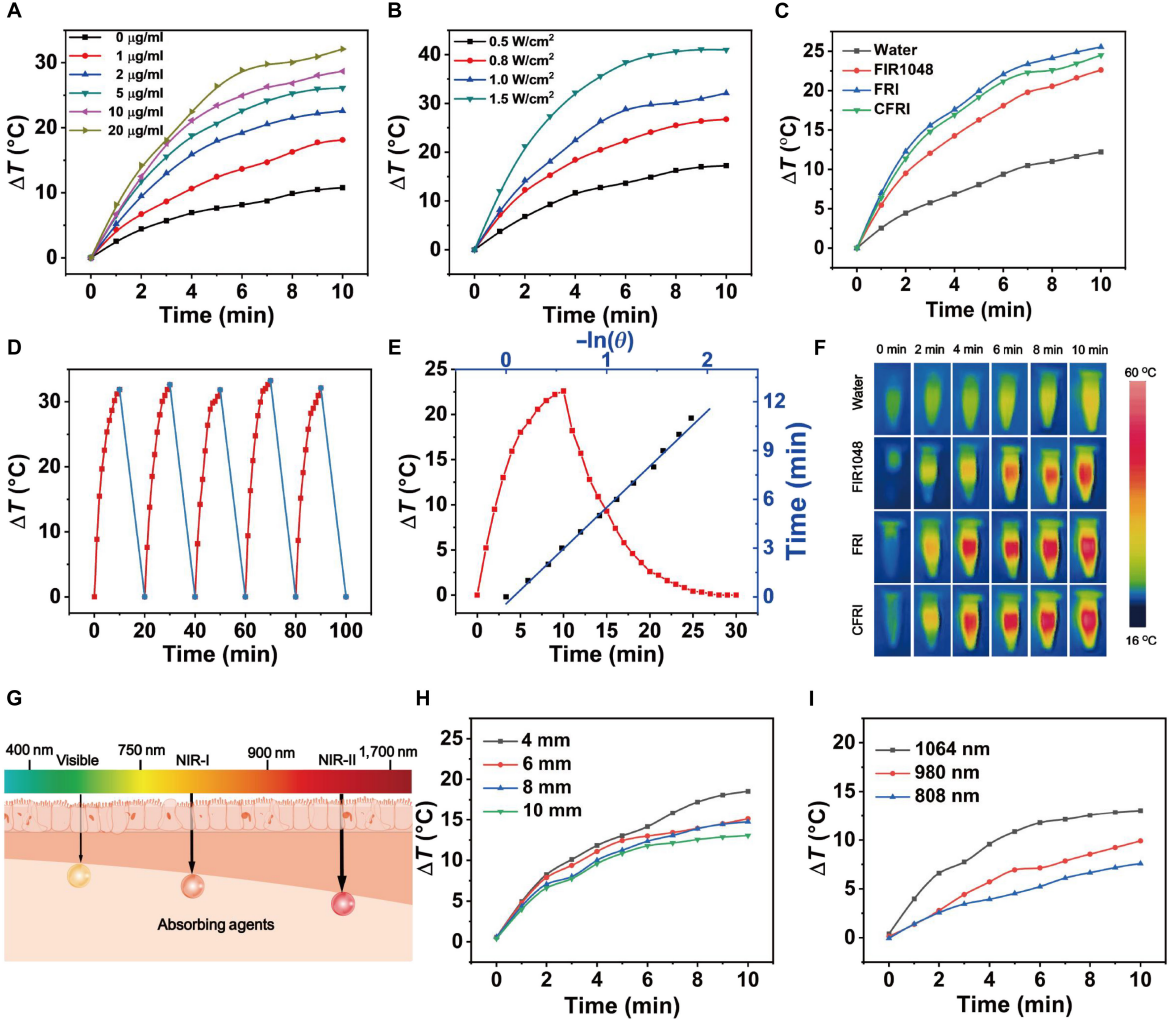
In vitro photothermal conversion performance. (A) Different concentrations (1, 2, 5, 10, and 20 μg/ml) under laser irradiation (1,064 nm, 1.0 W/cm^2^, 10 min). (B) At a concentration of 2 μg/ml (1,064 nm, 10 min), the temperature change curves at different laser power densities (0.5, 0.8, 1.0, and 1.5 W/cm^2^). (C) At a concentration of 2 μg/ml (1,064 nm, 1.0 W/cm^2^), the temperature change curves of water, FIR1048, FRI, and CFRI within 10 min. (D) CFRI (2 μg/ml) of aqueous solution under 1,064-nm laser irradiation (1.0 W/cm^2^, 10 min per cycle). (E) The linear relationship between CFRI cooling cycle and ln(*θ*). (F) Laser irradiation (1,064 nm, 1.0 W/cm^2^) had an effect on water, FIR1048, FRI, and CFRI (2 μg/ml) at different time points (0, 2, 4, 6, 8, and 10 min). (G) Laser penetration schematic diagram. (H) Temperature changes of CFRI nanoparticles (2 μg/ml) under different thicknesses of chicken breast meat (4, 6, 8, and 10 mm). (I) Temperature rise of the same concentration of photothermal agent under different laser irradiation wavelengths (808, 980, and 1,064 nm).

In our meticulous examination of the photothermal properties of CFRI nanoparticles, we utilized state-of-the-art real-time IR thermal imaging to meticulously assess the photothermal conversion efficiency of CFRI nanoparticles under ex vivo conditions. Our experimental paradigm initially involved the assessment of temperature elevation in CFRI aqueous solutions of varying concentrations upon irradiation with a 1,064-nm laser. The outcomes demonstrated a proportional rise in temperature with increasing concentrations of IR1048, underscoring a pronounced concentration-dependent dynamic (Fig. 3A). This insight suggests that by precisely adjusting the concentration of CFRI nanoparticles, we can effectively modulate the photothermal conversion efficacy, offering a viable approach for targeted thermal ablation of tumor cells. In subsequent investigative stages, with the CFRI nanoparticle concentration fixed at 2 μg/ml, we scrutinized the temperature variation profiles under a spectrum of laser power densities. The dataset indicated a direct correlation between the temperature increase of CFRI nanoparticles and the laser power, illustrating an exemplary power dependency (Fig. 3B). With a commitment to treatment safety, we prioritized temperature control during laser irradiation. Through exhaustive analysis of temperature profiles, we delineated the optimal CFRI nanoparticle concentration and laser power parameters to achieve effective thermal ablation of cancer cells while minimizing collateral impact on adjacent healthy tissues. Under the optimized parameters (CFRI nanoparticle concentration: 2 μg/ml; laser power: 1,064 nm, 1.0 W/cm^2^), FRI nanoparticles loaded with the immunoadjuvant R837 and those without (FIR1048) exhibited parallel temperature escalation curves, indicating that the integration of the immunoadjuvant did not significantly compromise photothermal conversion efficiency. Furthermore, CFRI nanoparticles adorned with the 4T1 cell membrane and their uncoated FRI counterparts displayed congruent temperature escalation patterns, signifying that the cell membrane coating did not decrease the photothermal efficacy (Fig. 3C). The photothermal stability of CFRI nanoparticles was further confirmed by their consistent performance across multiple heating and cooling cycles (Fig. 3D). The maximum temperature did not change by more than 1.5 °C in 5 repeated heating and cooling cycles, and the UV absorption curve did not change after each heating and cooling cycle, and the absorbance at the highest absorption did not change clearly. The CFRI nanoparticles maintained consistent photothermal properties after multiple cycles, demonstrating their reliability for practical applications (Fig. S2). Moreover, by employing a precise calculation correlating linear time data with −ln*θ*, we determined that the photothermal conversion efficiency of CFRI nanoparticles is remarkably high at 48.99% (Fig. 3E), positioning it as a leading contender among contemporary PTAs. To appraise the clinical applicability of CFRI nanoparticles, we extended our investigation to include an assessment of the tissue penetration capabilities of NIR-II lasers. Employing chicken breast of varying thicknesses as a proxy for biological tissue, we observed that even at a substantial depth of 10 mm, the CFRI-induced temperature elevation within a 10-min interval was nearly 13 °C (Fig. 3H), highlighting the exceptional heat transfer efficacy of NIR-II lasers in penetrating deep tissue strata. Additionally, comparative analysis with other NIR laser wavelengths under identical conditions revealed that the 1,064-nm laser induced the most substantial temperature elevation, further substantiating its superior tissue penetration attributes (Fig. 3I).

### In vitro cellular internalization of CFRI nanoparticles

**Fig. 4.**
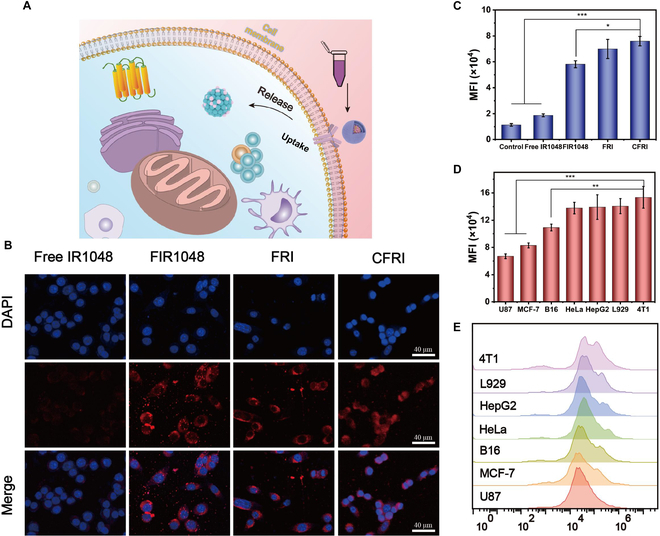
In vitro intracellular uptake of CFRI nanoparticles. (A) Schematic diagram of material uptake. (B) Confocal laser scanning microscopy (CLSM) images of 4T1 cells treated with free IR1048, FIR1048, FRI, or CFRI. Nuclei were stained blue with 4′,6-diamidino-2-phenylindole (DAPI) (scale bar = 40 μm). (C) The average fluorescence intensity of 4T1 cells by flow cytometry (co-cultured with free IR1048, FIR1048, FRI, or CFRI for 4 h). (D) The average fluorescence intensity of 4T1, MCF-7, B16, HeLa, HepG2, L929, and U87 cells after each incubation with CFRI nanoparticles for 4 h. (E) Flow cytometry histograms of 4T1, MCF-7, B16, HeLa, HepG2, L929, and U87 cells after each being incubated with CFRI nanoparticles for 4 h (*P < 0.05, **P < 0.01, and ***P < 0.001). MFI, mean fluorescence intensity.

We assessed the internalization efficiency of IR1048 in its diverse presentations (free, FIR1048, FRI, and CFRI) within the context of 4T1 breast cancer cells. Post coincubation of the 4T1 cells with the materials under investigation and nuclear staining with DAPI, we employed CLSM to meticulously examine the intracellular distribution of these materials. The colocalization analysis from CLSM imaging revealed a marked observation: CFRI nanoparticles demonstrated a pronounced degree of colocalization with the cell nuclei, with the red fluorescence signal of the nanoparticles tightly juxtaposed with the blue fluorescence delineation of the cell nuclei. This finding robustly confirms the exceptional endocytic capabilities of CFRI nanoparticles, illustrating their proficiency in efficiently transversing the cellular membrane and accumulating within the nuclei of 4T1 cells (Fig. 4A and B). Quantitative analysis performed using flow cytometry further validated the observations. The fluorescence signal intensity within the CFRI group was substantially elevated, reaching a notable 4.05-fold increase over the free IR1048 group. This disparity not only corroborates the substantial efficacy of the cell membrane coating strategy in augmenting cellular uptake efficiency but also alludes to the potential for intracellular drug accumulation, signifying promising therapeutic implications (Fig. 4C and Fig. S3). To penetrate deeper into the homologous targeting specificity of CFRI nanoparticles, we expanded our investigative scope to include a diverse array of human and murine tumor cell lines, such as MCF-7, B16, HeLa, HepG2, L929, and U87 cells. The results consistently indicated that CFRI exhibited the most robust fluorescence and highest permeability specifically in 4T1 cells. This enhanced targeting is likely attributed to the homologous targeting properties conferred by the 4T1 cancer cell membrane coating, as depicted in Fig. 4D and E. This discovery particularly accentuates the targeted homing propensity of CFRI nanoparticles toward their homologous tumor cells, providing substantial scientific evidence for the precision delivery of therapeutics and underscoring the potential for targeted cancer therapy.

### In vitro photothermal cytotoxicity of CFRI nanoparticles

**Fig. 5.**
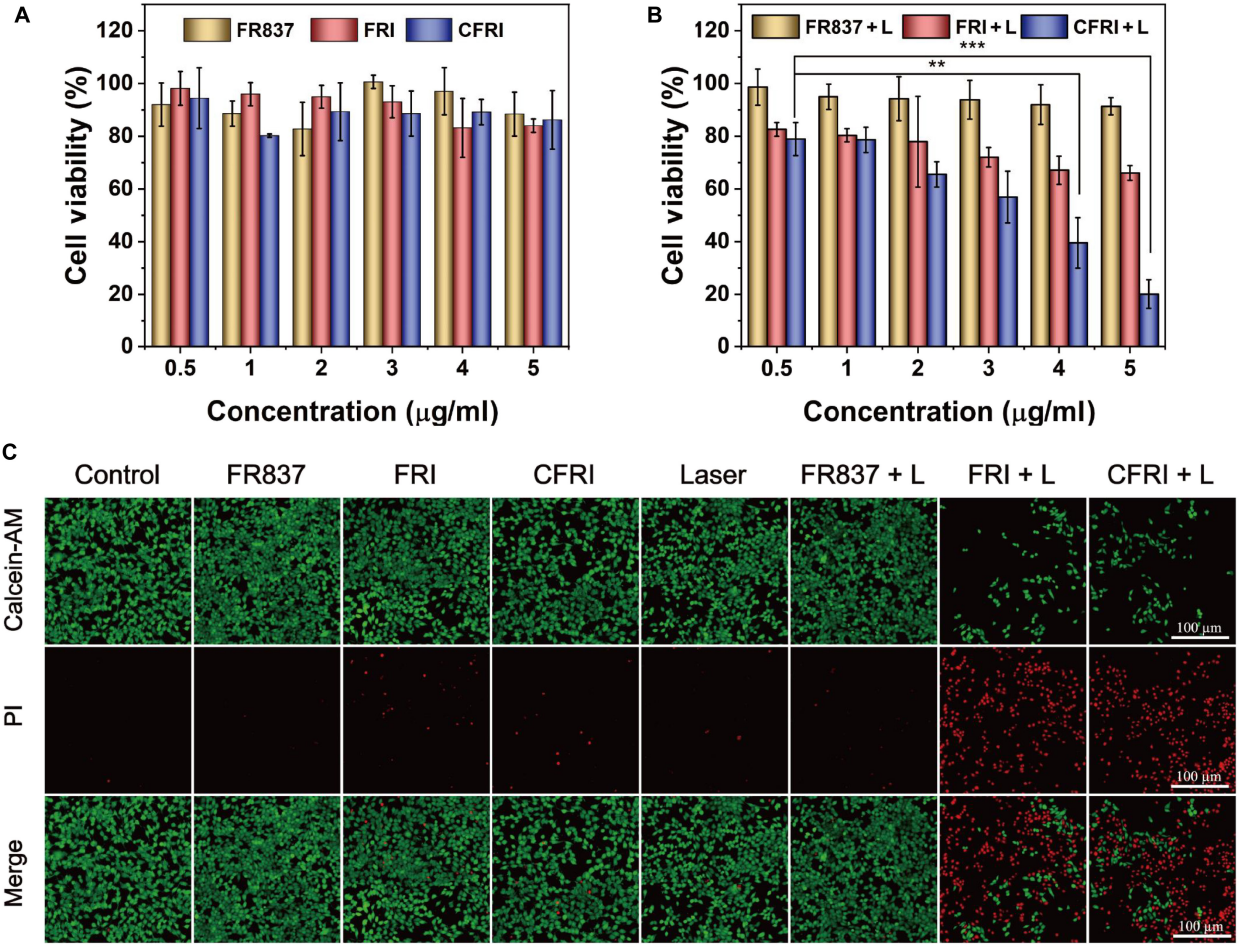
In vitro photothermal cytotoxicity of CFRI nanoparticles. (A) Cell viability after FR837, FRI, or CFRI treatment of 4T1 cells. (B) Cell viability of 4T1 cells treated with FR837, FRI or CFRI under 1,064-nm laser irradiation (1.0 W/cm^2^, 2 min). (C) Fluorescence images of 4T1 cells treated with Dulbecco’s modified Eagle medium (DMEM), FR837, FRI, or CFRI or irradiated by 1,064-nm laser (1.0 W/cm^2^, 2 min). Live cells were stained green with calcein-AM, and dead cells were stained red with propidium iodide (PI) (scale bar = 100 μm) (**P < 0.01 and ***P < 0.001).

In our meticulous investigation into the in vitro photothermal cytotoxic effects of CFRI nanoparticles, we utilized the MTT assay to meticulously evaluate the cytotoxic potential of CFRI nanoparticles. The experimental outcomes revealed that 4T1 cells, when co-cultured with CFRI nanoparticles for 24 h in the absence of laser illumination, maintained cellular viability above 90%. This finding not only confirms the superior biocompatibility of CFRI nanoparticles within a safe concentration range but also provides a solid foundation for subsequent PTT experiments (Fig. 5A). We then proceeded to expose 4T1 cells from both the FRI and CFRI groups to 1,064-nm laser irradiation for 2 min. The results elucidated that R837, classified as an immunosuppressive agent, did not exert toxicity on cancer cells, irrespective of their exposure to laser irradiation, indicating a lack of direct cytotoxic effect on cancer cells in vitro. However, as the concentration of IR1048 progressively increased, a pronounced decline in cell viability was observed, a trend that was closely correlated with the elevated photothermal conversion efficiency. Notably, when the IR1048 concentration was elevated to 5 μg/ml, the survival rate of cells treated with CFRI nanoparticles under laser irradiation was sharply reduced compared to that of the uncoated FRI group, with the survival rate experiencing a precipitous drop from 66.04% to below 20%. This marked discrepancy highlights the considerable advantage of cell-membrane-coated CFRI nanoparticles in PTT. By enhancing the cellular uptake of nanoparticles, it augments intracellular drug accumulation, thereby achieving efficient thermal ablation of tumor cells upon laser irradiation (Fig. 5B). To visually delineate the photothermal cytotoxicity of CFRI nanoparticles, we employed calcein-AM (green) and PI (red) fluorescence staining to distinguish between viable and nonviable cells. The microscopic images captured the thermal ablation effect of CFRI nanoparticles on 4T1 cells under laser irradiation, with a stark contrast between the distribution of viable and nonviable cells, vividly illustrating the cytotoxic effects of CFRI nanoparticles (Fig. 5C). Furthermore, using annexin V–FITC and PI dual staining, in tandem with flow cytometry analysis, we conducted an in-depth examination of the impact of CFRI nanoparticles on cellular apoptosis. The results demonstrated a marked rise in the proportion of cells undergoing late apoptosis, increasing from 38.9% to 54.5%, after treatment with CFRI nanoparticles and subsequent laser irradiation, further substantiating the substantial apoptotic impact of CFRI nanoparticles on cancer cells within the realm of PTT (Fig. S4).

### In vitro assessment of ICD effects

**Fig. 6.**
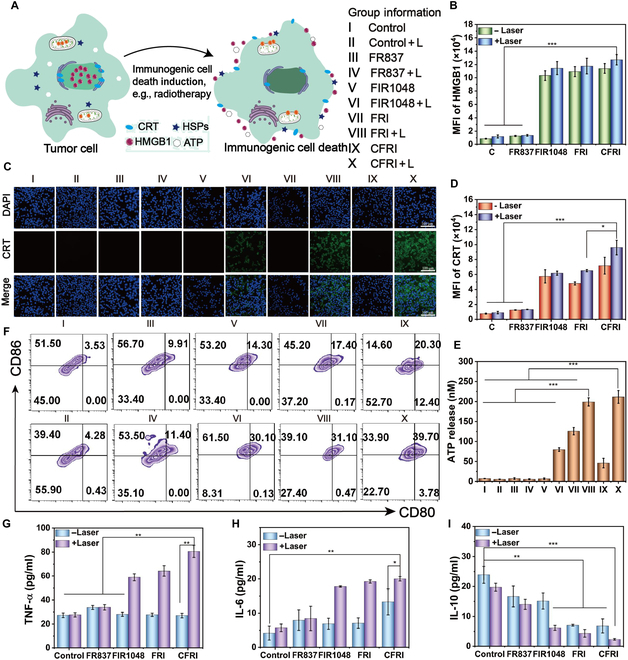
(A) CFRI-mediated ICD and DCs matured in vitro. Mechanism scheme of PTT-induced immunogenic death; 1,064-nm laser irradiation (1.0 W/cm^2^) with or without treatment with DMEM, FR837, FIR1048, FRI, and CFRI (2 μg/ml). (B) The efflux of HMGB1 was quantitatively analyzed by flow cytometry. (C) CLSM images of CRT expression on the 4T1 cell surface after different treatments (scale bar: 100 μm). (D) Mean fluorescence intensity of CRT expression in 4T1 cells after different treatments. (E) The average fluorescence intensity of ATP exposure in 4T1 cells after different treatments. (F) The corresponding quantification (CD80^+^CD86^+^) of mature DCs was quantitatively analyzed by flow cytometry. (G) After different treatments by enzyme-linked immunosorbent assay (ELISA), secretion levels of TNF-ɑ in the cell supernatant. (H) After different treatments by ELISA, the secretion level of IL-6 in the cell supernatant. (I) After different treatments by ELISA, the secretion level of IL-10 in the cell supernatant. Information of each group: I, control; II, control + L; III, FR837; IV, FR837 + L; V, FIR1048; VI, FIR1048 + L; VII, FRI; VII, FRI + L; IX, CFRI; X, CFRI + L. Significance is determined using one-way analysis of variance (*P < 0.05, **P < 0.01, and ***P < 0.001). HSPs, heat shock proteins.

In our rigorous investigation into the ICD effects induced by CFRI nanoparticles, we meticulously crafted and executed a comprehensive suite of experiments to evaluate the impact of CFRI nanoparticles on the immunogenic demise of tumor cells in vitro [29,30]. ICD, a novel mechanism for antitumor immune responses, is critical for its capacity to induce the release of specific DAMPs from tumor cells under certain stimuli, thereby initiating an immune system assault on the tumor (Fig. 6A). In the previous section, MTT assay was used to determine the IC_50_ concentration (2 μg/ml) at which CFRI nanoparticles could effectively induce cell death without causing excessive toxicity. Concentrations below IC_50_ may not be sufficient to cause cell death, whereas concentrations above IC_50_, although likely to induce more potent cytotoxic and apoptotic effects, may also increase nonspecific cell death at the same time. Therefore, the IC_50_ of CFRI (2 μg/ml) was selected for ICD induction. To further explore the effect of CFRI on ICD induction, we selected concentrations near IC_50_ (0, 1, 3, and 5 μg/ml) to explore the secretion of HMGB1 and CRT after light treatment by flow analysis. The results showed that the highest levels of HMGB1 and CRT were found at 3 μg/ml of CFRI, indicating that the higher the concentration of CFRI, the better the effect of ICD induction. Although a high concentration can cause stronger cytotoxicity and apoptosis effects, it may also increase nonspecific cell death and not necessarily induce ICD (Fig. S5). Experimental findings revealed that CFRI nanoparticles, under 1,064-nm laser irradiation, can efficiently initiate the ICD process in 4T1 cells. Data from immunofluorescence analyses demonstrated that the release of HMGB1 in cells treated with CFRI nanoparticles in conjunction with laser irradiation (CFRI + L group) was markedly pronounced, with the fluorescence intensity marked surpassing that of other treatment groups (Fig. 6B and Fig. S6). This discovery underscores the exceptional efficacy of CFRI nanoparticles (2 μg/ml) in inducing ICD. Moreover, observations through CLSM and flow cytometry indicated a sharp increase in the expression of CRT on the surface of 4T1 cells treated with the CFRI + L group, a key indicator of successful ICD induction (Fig. 6C and D). The fluorescence intensity of CRT expression induced by the CFRI group was substantially higher than those of other groups, with the CFRI + L group exhibiting a 10.4-fold increase over the control + L group, a 7.0-fold increase over the FR837 + L group, and a 1.6-fold increase over the FIR1048 + L group. Notably, compared to the uncoated FRI group, the CFRI (2 μg/ml) group, which employed cell membrane encapsulation, induced a higher level of CRT, as confirmed by CLSM images. This result further substantiates the important role of the cell membrane encapsulation strategy in enhancing the immunogenic properties of CFRI nanoparticles. In experiments of monitoring ATP release levels, we also observed a consistent trend between the amount of ATP released by CFRI nanoparticles and the expression levels of CRT and HMGB1 release, once again confirming the effectiveness of CFRI nanoparticles in inducing ICD effects (Fig. 6E). Collectively, these experimental results converge on a conclusion: the photothermal effect induced by laser irradiation of the PTA can stimulate the expression and release of HMGB1, CRT, and ATP, activating the ICD pathway and thus effectively enhancing the immune response.

Furthermore, the ATP release, CRT exposure, and HMGB1 extrusion induced by the photothermal effect increase the exposure of DAMPs and activate the ICD pathway, which in turn promotes the maturation of DCs. To gain an in-depth understanding of the impact of CFRI-mediated ICD on DC maturation, we employed flow cytometry to analyze the proportion of mature DCs (CD80^+^CD86^+^). The results showed that the maturation rate of DCs in the CFRI + L group increased from 4.28% to 39.70% compared to that of the control + L group. Moreover, when compared to the maturation rate for the CFRI group without irradiation, the maturation rate of DCs also increased by 19.40%, a change closely associated with the ICD effect induced by CFRI nanoparticles (Fig. 6F). The trend in DC maturation was congruent with that of the ICD activation pathways represented by ATP release, CRT exposure, and HMGB1 extrusion, indicating that photothermal immunotherapy mediated by CFRI + L can increase DAMP exposure and promote DC cell maturation through the ICD mechanism. Compared to single-agent IR1048-mediated PTT or R837-induced monotherapy, the material coloaded with the PTA IR1048 and the immunoadjuvant R837 can induce a higher rate of DC maturation and enhance the immune response through the exposure of DAMPs induced by PTT and the adjuvant function of the immunoadjuvant. When used in conjunction with immunotherapy, PTT plays a crucial role in inhibiting the transfer and recurrence of tumors in vivo. We analyzed the cytokines in the supernatants of different treatment groups using ELISA. We examined 3 key cytokines: TNF-α (a cytokine for cellular immune activation), IL-6 (a marker for the activation and proliferation of immune cells), and IL-10 (a regulatory cytokine that suppresses immune responses through contact-dependent inhibition). Compared to other groups, the supernatants of cells treated with the CFRI + L group exhibited higher levels of the pro-inflammatory cytokines TNF-α and IL-6, while the level of the anti-inflammatory cytokine IL-10 was lower, indicating that CFRI-mediated photothermal immunotherapy can effectively activate immune cells and enhance antitumor immune responses (Fig. 6G to I).

### In vivo imaging facilitated by CFRI nanoparticles

Within the field of oncological treatment, the localization and retention of therapeutic agents at the tumor site are essential for therapeutic success. To meticulously track the dynamic distribution and metabolic behavior of these agents in vivo, we employed the NIR-II fluorescent dye IR1048. Utilizing the exceptional fluorescence characteristics of IR1048, we enabled the real-time monitoring of CFRI nanoparticles within the biological system, eliminating the necessity for supplementary fluorescent markers. In our evaluation of the in vivo targeting efficacy of CFRI nanoparticles, we administered both cell-membrane-modified and unmodified CFRI nanoparticles into 4T1 tumor-bearing mice models via tail vein injection. The experimental results demonstrated that the fluorescence signal in the CFRI group rapidly accumulated at the tumor site, peaking after 60 h and exceeding the intensity of the uncoated FRI group. This observation suggests that the incorporation of the cell membrane enhances the tumor-targeting specificity of CFRI nanoparticles (Fig. S7). To gain a deeper understanding of the circulatory properties of CFRI nanoparticles in vivo, we performed an in situ injection at the tumor site, followed by an extended observation period of their fluorescence signal persistence. Notably, the fluorescence signal at the tumor site remained detectable even after 120 h, indicating that CFRI nanoparticles possess an extended circulation time in vivo. This characteristic provides a robust foundation for the sustained release of therapeutic agents and the maintenance of therapeutic efficacy in cancer treatment (Fig. S8). In addition, photoacoustic imaging of mouse tumor sites was performed using FIR1048 and CFRI, both of which rapidly accumulated to the tumor site within 6 h, facilitating the simulation of tumor contours and facilitating tumor localization. Moreover, at 6 h, the photoacoustic signal of CFRI was much higher than that of FIR1048, indicating that the bionic nanoparticles had a good photoacoustic effect (Fig. S9).

**Fig. 7.**
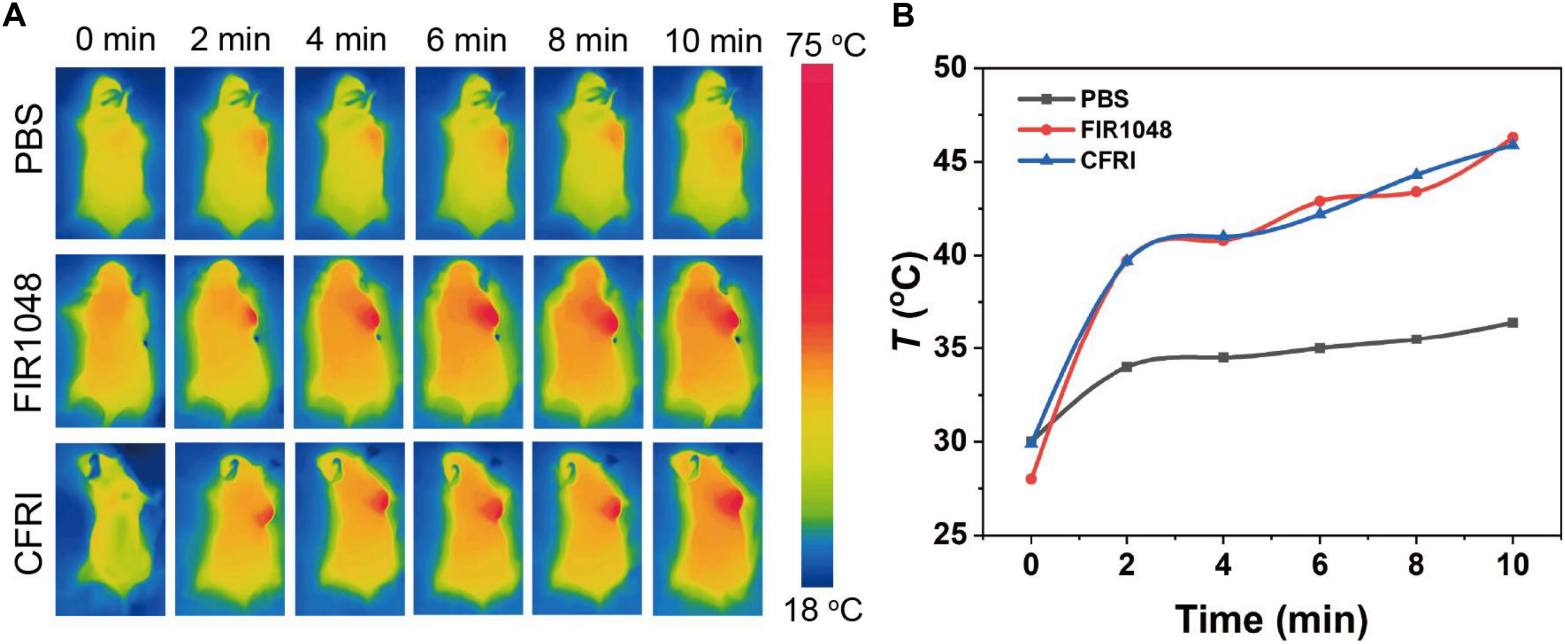
(A) 4T1 tumor-bearing mice were irradiated by injection of phosphate-buffered saline (PBS), FIR1048, and CFRI under 1,064-nm laser (1.0 W/cm^2^, 10 min). (B) Under 1,064-nm laser irradiation, the temperature change curve of tumor within 10 min.

In the assessment of in vivo photothermal effects, we intravenously injected PBS, FIR1048, or CFRI materials into mice for 4 h; the mice were then subjected to 1,064-nm laser irradiation to monitor real-time changes in tumor temperature using an IR thermal instrument. As depicted in Fig. 7A and B, within the span of 10 min under laser irradiation, the tumor temperature of mice administered with FIR1048 escalated from 28 to 48 °C, while the tumor temperature of mice injected with CFRI nanoparticles rose from 30 to 47 °C. This phenomenon confirms the high efficiency of CFRI nanoparticles in converting NIR light into thermal energy for the thermal ablation of solid tumors. The temperature monitoring of normal tissues around the tumor in mice (Fig. S10) showed that the temperature did not reach 40 °C, indicating that photothermal treatment with CFRI did not cause the death of surrounding normal cells.

### In vivo antitumor efficacy of CFRI nanoparticles

**Fig. 8.**
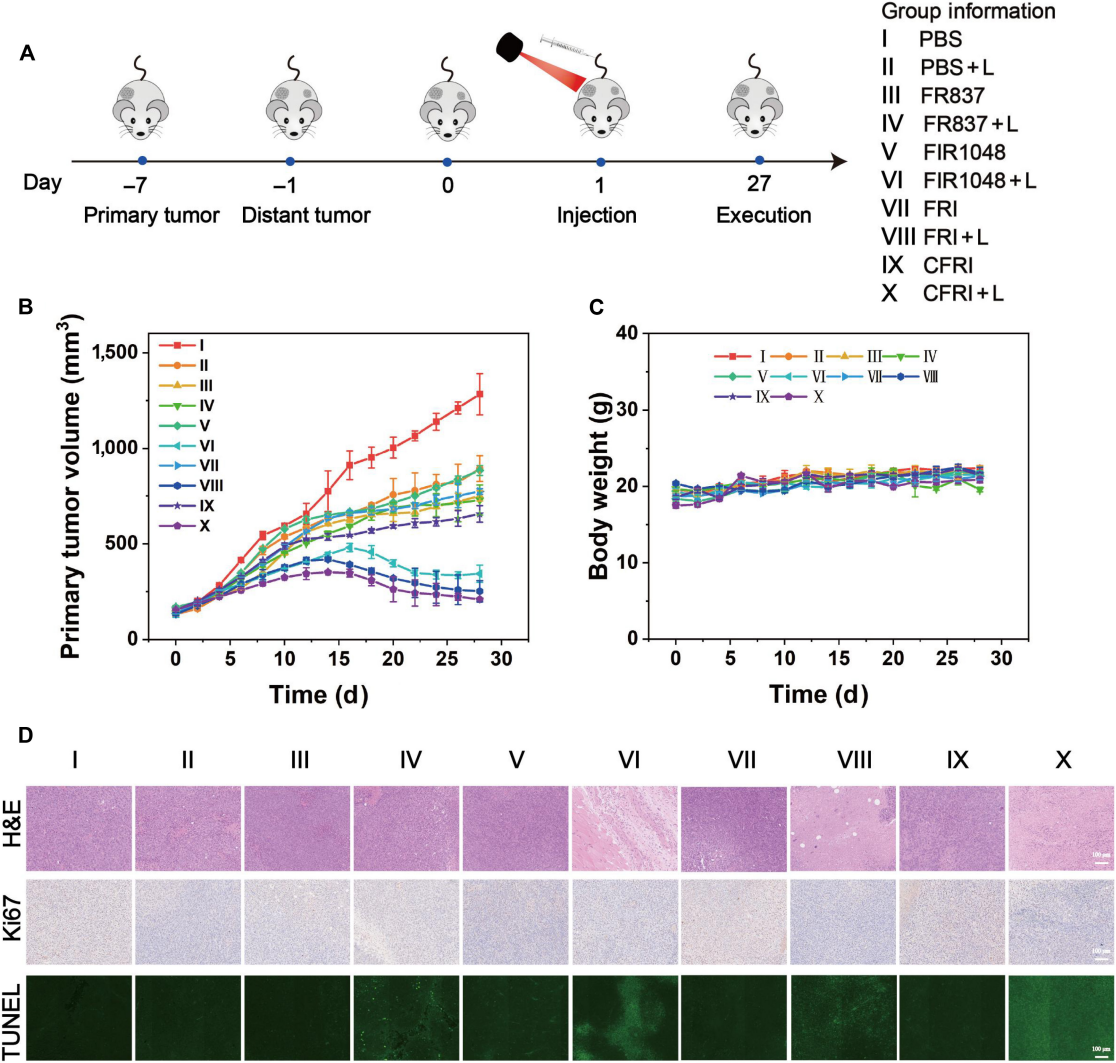
CFRI-mediated photothermal synergistic immunotherapy. (A) Schematic diagram of the establishment of a bilateral 4T1 tumor model in mice. (B) The tumor volume of the primary tumor after treatment in different groups of 4T1 tumor-bearing mice (PBS, FR837, FIR1048, FRI, or CFRI with or without laser irradiation [1,064 nm, 1.0 W/cm^2^, 2 min]). (C) Weight curves of 4T1 tumor-bearing mice after different treatments. (D) Hematoxylin and eosin (H&E), terminal deoxynucleotidyl transferase-mediated dUTP nick-end labeling (TUNEL), and Ki67 staining images of 4T1 tumor-bearing mice after different treatments. Scale bar: 100 μm. Group information: I, PBS; II, PBS + L; III, FR837; IV, FR837 + L; V, FIR1048; VI, FIR1048 + L; VII, FRI; VIII, FRI + L; IX, CFRI; X, CFRI + L.

In our exhaustive examination of the in vivo antineoplastic effects of CFRI nanoparticles, we utilized a bilateral 4T1 tumor model that adeptly emulates the clinical context of both primary and metastatic tumor lesions. Through a meticulously devised therapeutic strategy, which involved the intravenous administration of various pharmaceutical agents coupled with 1,064-nm laser irradiation, we systematically executed a synergistic photothermal and immunotherapy regimen targeting the mice’s primary tumors (Fig. 8A). Specifically, female BALB/c mice were administered a spectrum of treatments via intravenous injection (I, control group; II, control group + laser; III, FR837; IV, FR837 + laser; V, FIR1048; VI, FIR1048 + laser; VII, FRI; VIII, FRI + laser; IX, CFRI; X. CFRI + laser), with laser irradiation applied every 3 d. The primary tumor was subjected to 1,064-nm laser (1.0 W/cm^2^) irradiation after 4 h postinjection, while the distant tumor remained unaltered. To visually assess the therapeutic outcomes, we documented posttreatment images of the mice’s tumors (Fig. S11) and measured the tumor volumes. The results indicated that, with the exception of the PBS + L and FR837 + L groups, the growth rate of the primary tumors in the other laser-irradiated groups was marked curtailed, demonstrating the favorable suppressive effect of PTT on primary tumors and confirming the thermal ablation efficacy of PTT. Notably, after 27 d of treatment, the tumor volume in the CFRI + L group was considerably smaller than that of the other groups, suggesting that the treatment in the CFRI + L group nearly completely inhibited the growth of the primary tumor (Fig. 8B). As anticipated, the synergistic effect of photothermal treatment and immunotherapy enhanced the antineoplastic potency of the nanoparticles. Subsequently, the growth of distant tumors in mice was continuously observed and recorded. It was found that the volume change trend of the distal tumor in mice after different material treatments was consistent with that of the primary tumor, corroborating the previous conclusion. The distant tumor growth was inhibited to varying degrees in all laser irradiation groups except the control group, and the consistent effect of the CFRI + L group was the most sensible. This inhibition was attributed to the systemic immune response triggered by ICD induced by PTT. In addition, the most clear inhibition of distant tumors was observed with CFRI + L, as compared with FR837 or FIR1048 alone, indicating that the combination of PTT and immunotherapy obviously enhanced antitumor efficacy (Fig. S12).

To further substantiate the antineoplastic effects of CFRI nanoparticles, we performed H&E, Ki67, and TUNEL staining on the primary tumor tissues from each group. The H&E staining results revealed that the tumor cells in the PBS, FR837, FIR1048, FRI, and CFRI groups were orderly arranged and tightly structured, with no apparent damage observed. In contrast, tumor sections from the FIR1048 + L, FRI + L, and CFRI + L groups exhibited evident cellular destruction and nuclear pyknosis (Fig. 8D). This indicates that under NIR laser irradiation, CFRI nanoparticles demonstrated clear antineoplastic effects. Further investigation through TUNEL and Ki67 assays revealed that, compared to other groups, the CFRI + L group had the fewest proliferating cells and the highest number of apoptotic cells, highlighting the importance of PTT and immunosuppressive agents in tumor suppression. Consequently, CFRI-mediated photothermal immunotherapy not only suppresses the growth of primary tumors but also triggers a distant effect, delaying the growth of distant tumors.

In terms of biosafety evaluation, CFRI nanoparticles exhibited remarkable tolerability. During the treatment period, there were no visible changes in the body weight of mice across all groups, indicating the extremely low toxicity of CFRI nanoparticles in vivo (Fig. 8C). We collected plasma and tissues from major organs such as the heart, liver, spleen, lungs, and kidneys from the mice and conducted tissue, blood, biochemical, and pathological analyses. The blood routine and biochemical indicators of all groups of mice were within the normal range, indicating that the treatment did not cause liver or kidney dysfunction (Fig. S13). Additionally, H&E staining images showed no visible tissue damage to any major organs (Fig. S14). These results suggest that CFRI nanoparticles have good biocompatibility and safety in vivo, providing crucial preliminary data for their potential future clinical application.

### CFRI-mediated immune response

**Fig. 9.**
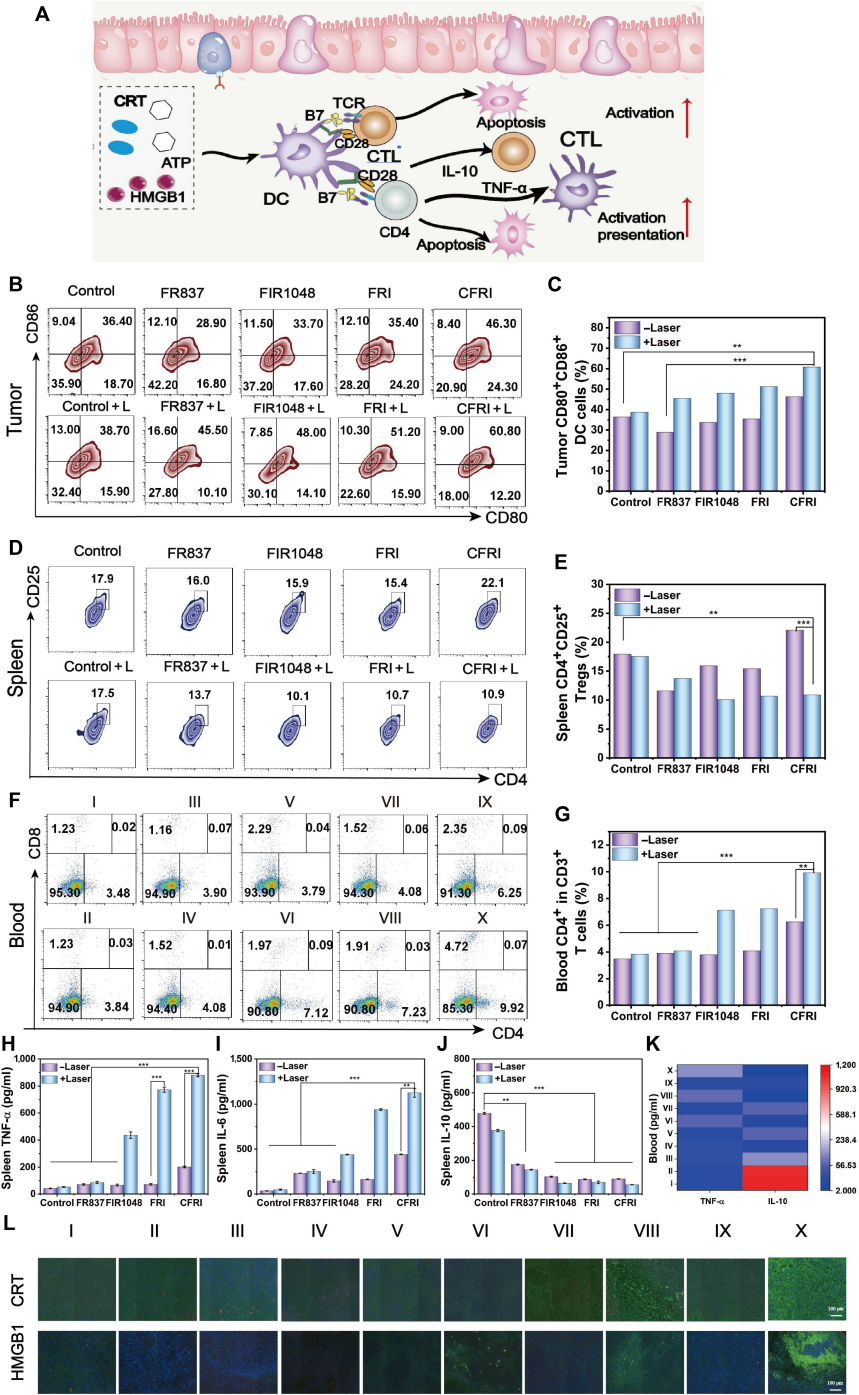
In vivo CFRI-mediated PTT-enhanced immunotherapy elicited an immune response after 16 d of treatment. (A) Schematic diagram of immunity induced by damage-associated molecular patterns (DAMPs). (B and C) Analysis of DC maturity (CD11^+^CD80^+^CD86^+^) using flow cytometry in the primary tumor of 4T1 tumor-bearing mice. (D and E) Regulatory T cells (Tregs) (CD4^+^CD25^+^) in the spleen. (F and G) T cell proliferation in the blood (CD4^+^CD8^+^). (H) The content of TNF-α in the spleen of 4T1 tumor-bearing mice. (I) The content of IL-6 in the spleen of 4T1 tumor-bearing mice. (J) The content of IL-10 in the spleen of 4T1 tumor-bearing mice. (K) Heat map of TNF-α and IL-10 content in the blood of 4T1 tumor-bearing mice. (L) CRT and HMGB1 staining images of 4T1 tumor-bearing mice after different treatments. Scale bar: 100 μm. Group information: I, PBS; II, PBS + L; III, FR837; IV, FR837 + L; V, FIR1048; VI, FIR1048 + L; VII, FRI; VIII, FRI + L; IX, CFRI; X, CFRI + L (*P < 0.05, **P < 0.01, and ***P < 0.001). CTL, cytotoxic T lymphocyte; TCR, T cell receptor.

To achieve a comprehensive understanding of the activation and modulation of the antitumor immune response by CFRI nanoparticles, key immunological indicators, such as DC maturation, T cell proliferation, and cytokine secretion profiles, were investigated. DCs, critical for initiating immune responses, displayed increased maturation in all laser-irradiated groups except the PBS control (Fig. 9A). The CFRI + L group showed the most obvious enhancement, rising from 38.7% to 60.8% (Fig. 9B and C), which indicates the potent effect of CFRI nanoparticles on DC maturation. Regulatory T cells, which suppress immune responses, were found to decrease in the CFRI + L group from 17.9% to 10.9%, suggesting a reduction in immunosuppression and a more robust immune response (Fig. 9D and E). CD4^+^ and CD8^+^ T lymphocytes, essential for antitumor immunity, showed markedly increased levels in the CFRI + L group, with a 2.85-fold and a 3.84-fold increase in blood levels, respectively (Fig. 9F and G and Fig. S15). Splenic infiltration of these T cells was also highest in the CFRI + L group, with CD4^+^ T cells increasing from 7.31% to 11.90% and CD8^+^ T cells from 2.68% to 4.84% (Fig. S16). This suggests that CFRI-mediated PTT evidently promotes the differentiation of immature T cells into CD4^+^ and CD8^+^ T cells, enhancing immune surveillance and tumor elimination and potentially preventing tumor progression, recurrence, or metastasis.

Pro-inflammatory cytokines IL-6 and TNF-α, positively associated with antitumor efficacy, and the anti-inflammatory cytokine IL-10, negatively associated, were measured by ELISA. The CFRI + L group showed higher levels of TNF-α and IL-6, and lower levels of IL-10, confirming the immunomodulatory role of CFRI nanoparticles in promoting pro-inflammatory responses and suppressing anti-inflammatory ones, thus amplifying the antitumor immune response (Fig. 9H to K). Immunofluorescence staining of tumor sections for HMGB1 and CRT expression revealed enhanced green fluorescence in the CFRI + L group, indicating the highest induced expression of CRT and consistent HMGB1 release, confirming the excellent effect of CFRI nanoparticles in inducing ICD effects (Fig. 9L). All of the above results demonstrated that CFRI-mediated photothermal immunotherapy effectively enhances immune surveillance and clearance of tumors by promoting DC maturation, T cell activation, and cytokine secretion modulation, presenting a promising therapeutic strategy for cancer treatment.

## Discussion

CFRI biomimetic nanoparticles, leveraging 4T1 cancer cell membranes to enhance tumor targeting and combining PTT with immunotherapy for effective cancer treatment, were successfully constructed. In vitro experiments demonstrated that CFRI biomimetic nanoparticles had high biocompatibility, photothermal efficiency, and improved cellular uptake, particularly under 1,064-nm laser irradiation, which induced ICD. In this process, the release of DAMPs is crucial for immune system activation against cancer. In vivo experiments further displayed that CFRI biomimetic nanoparticles showed excellent tumor targeting, circulation, and photothermal effects, effectively suppressing primary and metastatic tumor growth in mice. CFRI-mediated PTT and TLR7 agonist release promoted DC maturation, enhancing CD8^+^ and CD4^+^ T cell activity, key for tumor elimination and preventing metastasis. CFRI nanoparticles present a promising therapeutic platform for cancer eradication, offering new insights in oncology and potential for advanced cancer treatment development.

## Data Availability

The data used to support the findings of this work are available from the corresponding authors upon request.
