# Supplementary material for: Biomimetic-Nanoparticle-Enhanced Photothermal Immunotherapy: Targeted Delivery of Near-Infrared Region II Agents and Immunoadjuvants for Tumor Immunogenicity
Source: Biomater Res. 2025 Mar 4;29:0151. doi: 10.34133/bmr.0151 (PMC11876542; doi:10.34133/bmr.0151)
Supplement: Supplementary 1 — Figs. S1 to S16 [file bmr.0151.f1.pdf]

Supporting Information

Biomimetic Nanoparticles-Enhanced Photothermal Immunotherapy: Targeted Delivery of NIR-II Agents and Immunoadjuvants for Augmented Tumor Immunogenicity and Metastasis Suppression

Yanlu Yu,[1] Wen Li,[1] Qiqi Yu,[1] Jingtao Ye,[1] Hu Wang,[2] Yang Li,[1,] Shouchun Yin[1,]**

Y. Yu, W. Li, Q. Yu, J. Ye, Y. Li, S.Yin

[1]Key Laboratory of Organosilicon Chemistry and Materials Technology of Ministry of Education, College of Materials, Chemistry and Chemical Engineering, Hangzhou Normal University, 311121 Hangzhou, P. R. China

Email address: liyang@hznu.edu.cn; yinsc@hznu.edu.cn

H. Wang

[2]Key Laboratory of Ageing and Cancer Biology of Zhejiang Province, Institute of Ageing Research, School of Medicine, Hangzhou Normal Universtiy, 311121 Hangzhou, P. R. China

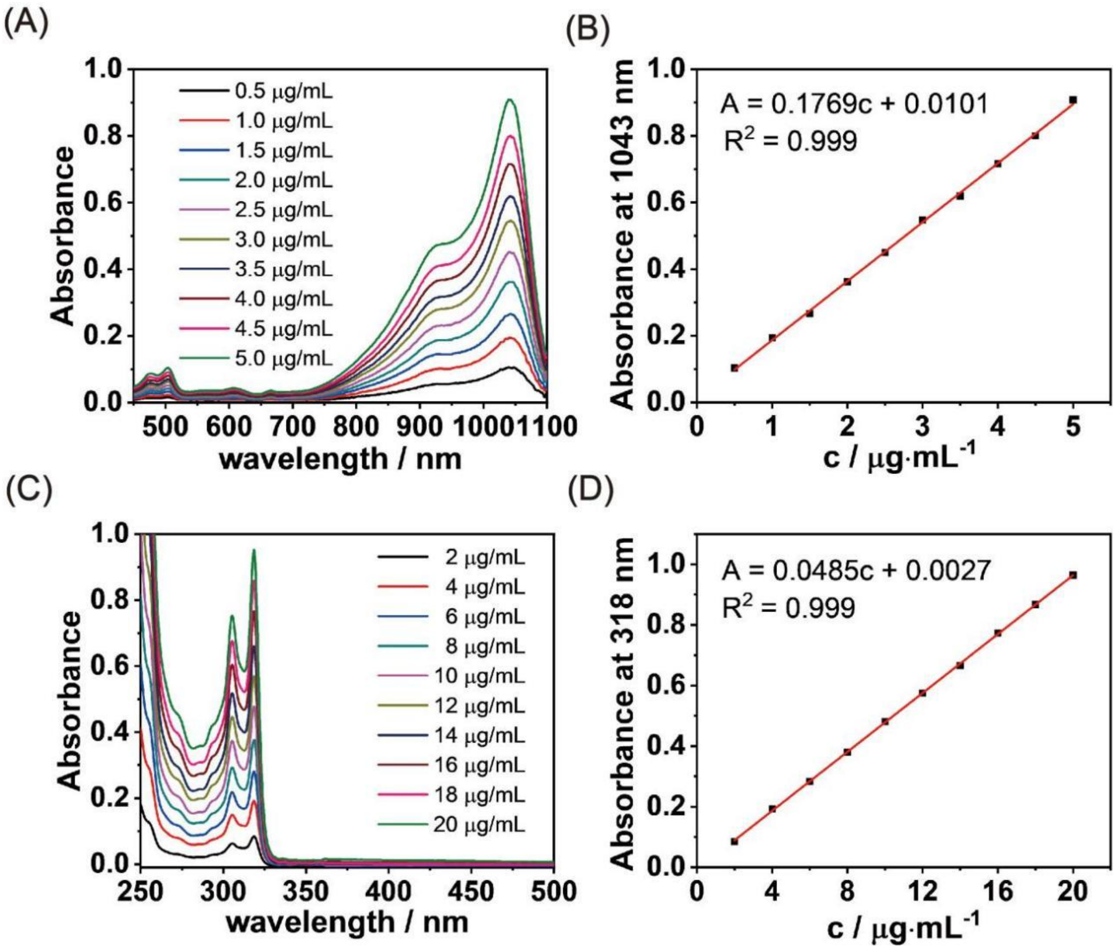

Fig. S1 (A) Absorption spectra and (B) standard curves of IR1048 in DMSO at various concentrations. (C) Absorption spectra and (D) standard curves of R837 in HAc at various concentrations.

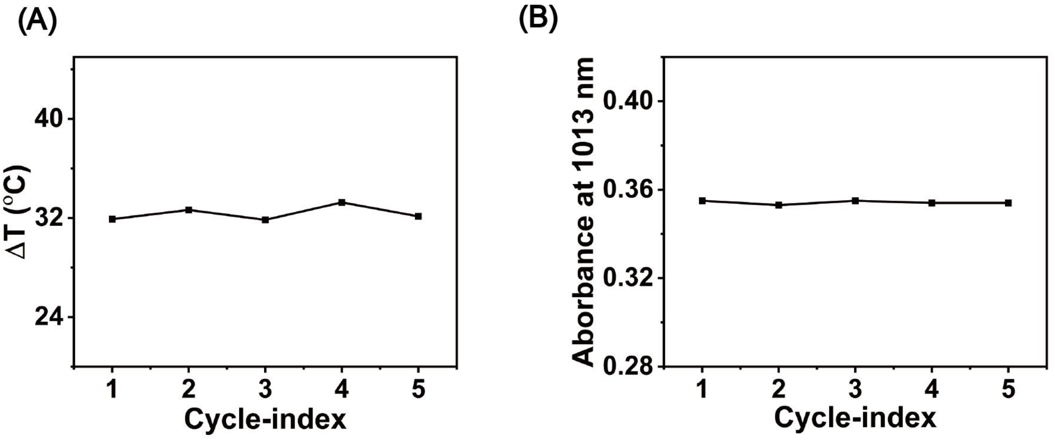

Fig. S2 (A) Maximum temperature variation curves of the five thermo-cooling cycles of CFRI. (B) Absorbance change curve of CFRI at 1013 nm after thermal cycling.

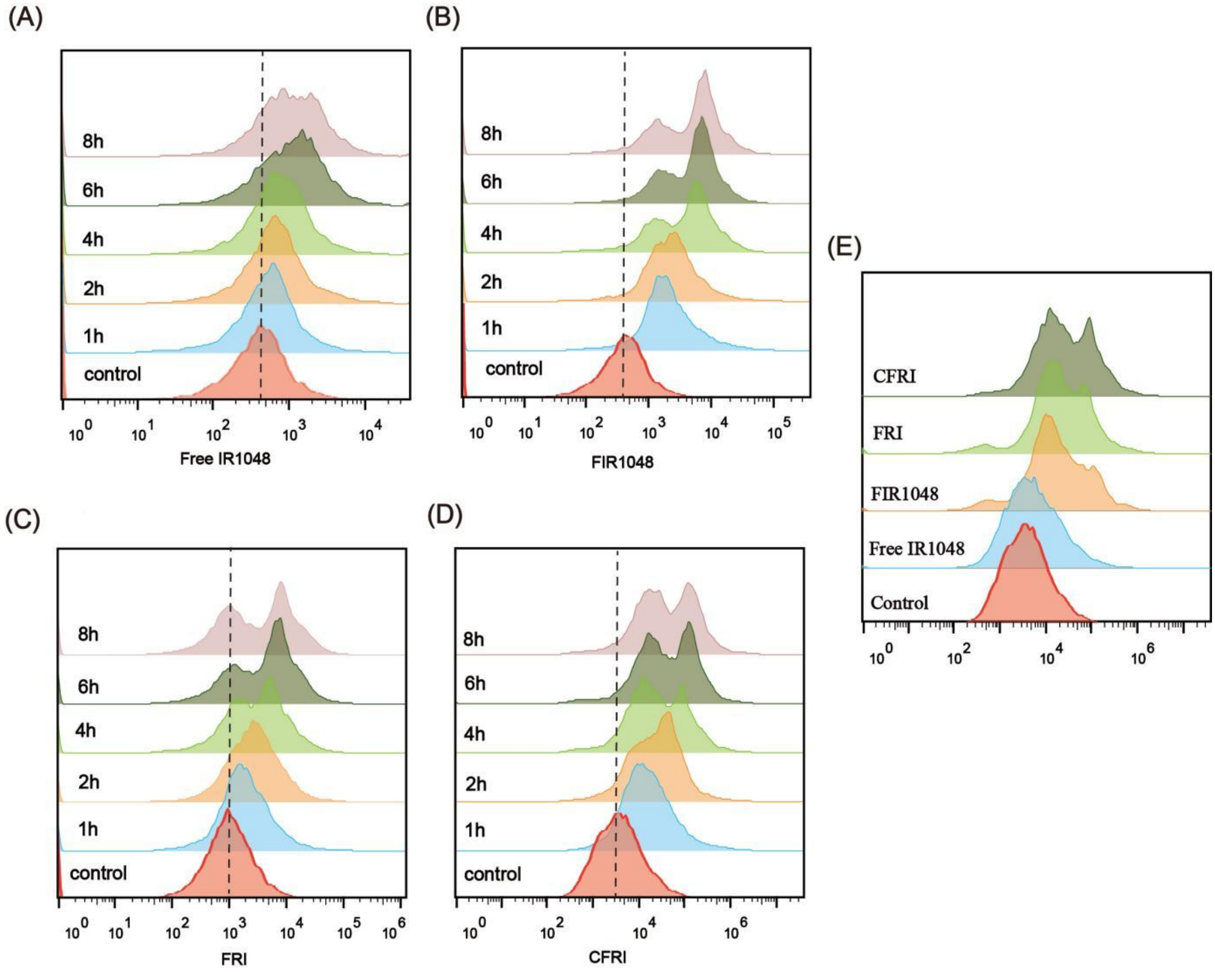

Fig. S3 (A) Flow cytometry of 4T1 cells incubated with free IR1048 for 8 h. (B) Flow cytometry of 4T1 cells incubated with FIR1048 for 8 h. (C) Flow cytometry of 4T1 cells incubated with FRI for 8 h. (D) Flow cytometry of 4T1 cells incubated with CFRI for 8 h. (E) Flow cytometry of 4T1 cells incubated with different materials (free IR1048, FIR1048, FRI, or CFRI) for 4 h.

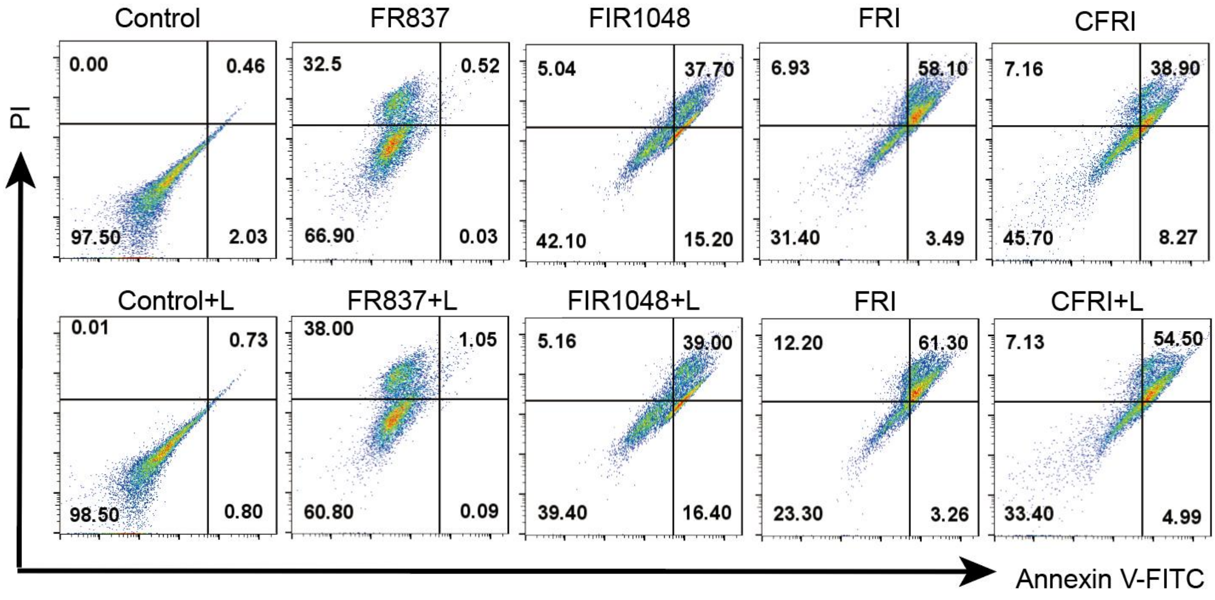

Fig. S4 Flow cytometry of 4T1 cell apoptosis treated with different drugs (Control, Control + L, FR837, FR837 + L, FIR1048, FIR1048 + L, FRI, FRI + L, CFRI, CFRI + L).

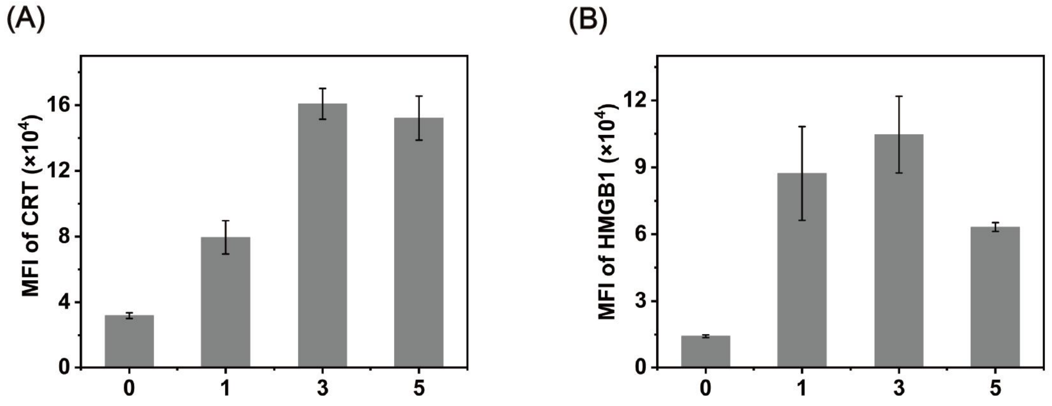

Fig. S5 (A) Flow cytometry was used to quantitatively analyze CRT efflux after treatment with different concentrations of CFRI (0, 1, 3, and 5 μg/mL). (B) Flow cytometry was used to quantitatively analyze HMGB1 efflux after treatment with different concentrations of CFRI (0, 1, 3, and 5 μg/mL).

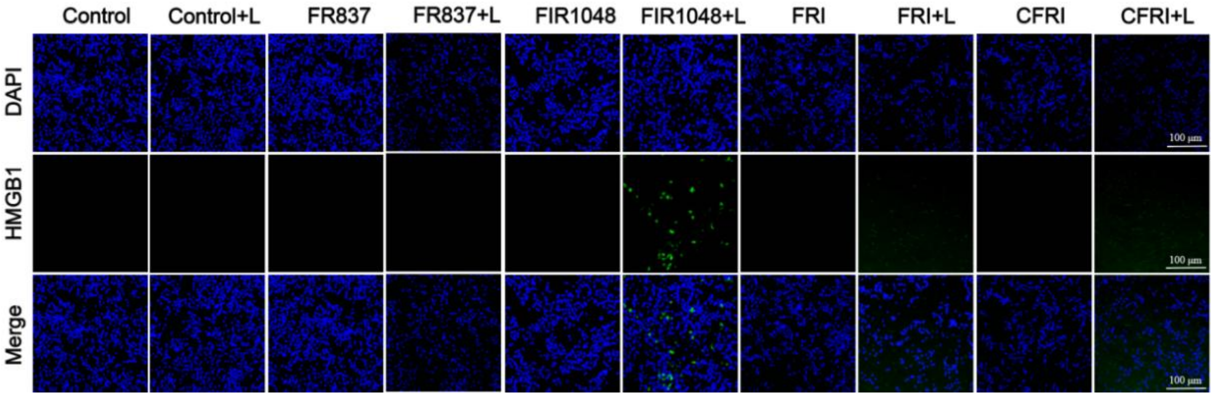

Fig. S6 Immunofluorescence images of HMGB1 exposure on the surface of 4T1 cells treated with different drugs (Control, Control + L, FR837, FR837 + L, FIR1048, FIR1048 + L, FRI, FRI + L, CFRI, CFRI + L).

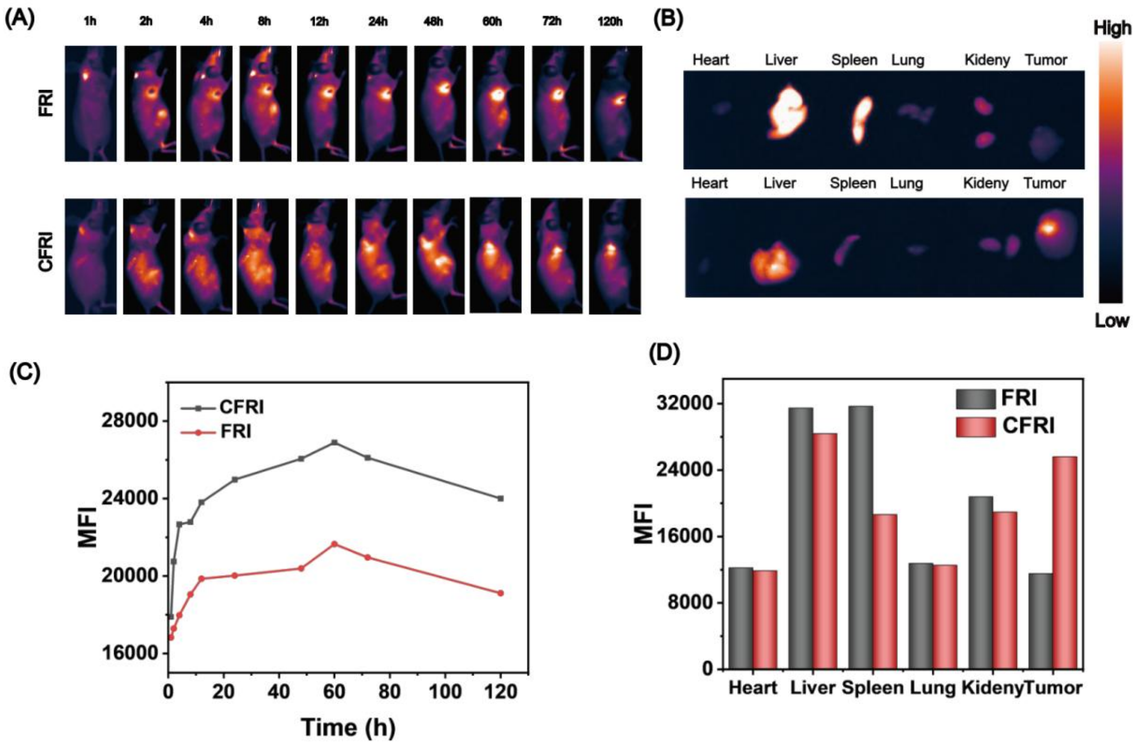

Fig. S7 *In vivo*, CFRI mediated biological distribution and photothermal imaging. (A) NIR fluorescence imaging of subcutaneous 4T1 tumor-bearing mice at different time points after tail vein injection of FIR1048 or CFRI. (B) *In vitro* fluorescence imaging of tumors and major organs (heart, liver, spleen, lung, kidney) in 4T1 tumor-bearing mice after intravenous injection for 120 h. (C) Quantitative analysis of the fluorescence intensity in 4T1 tumors. (D) The fluorescence intensity of tumors and major organs.

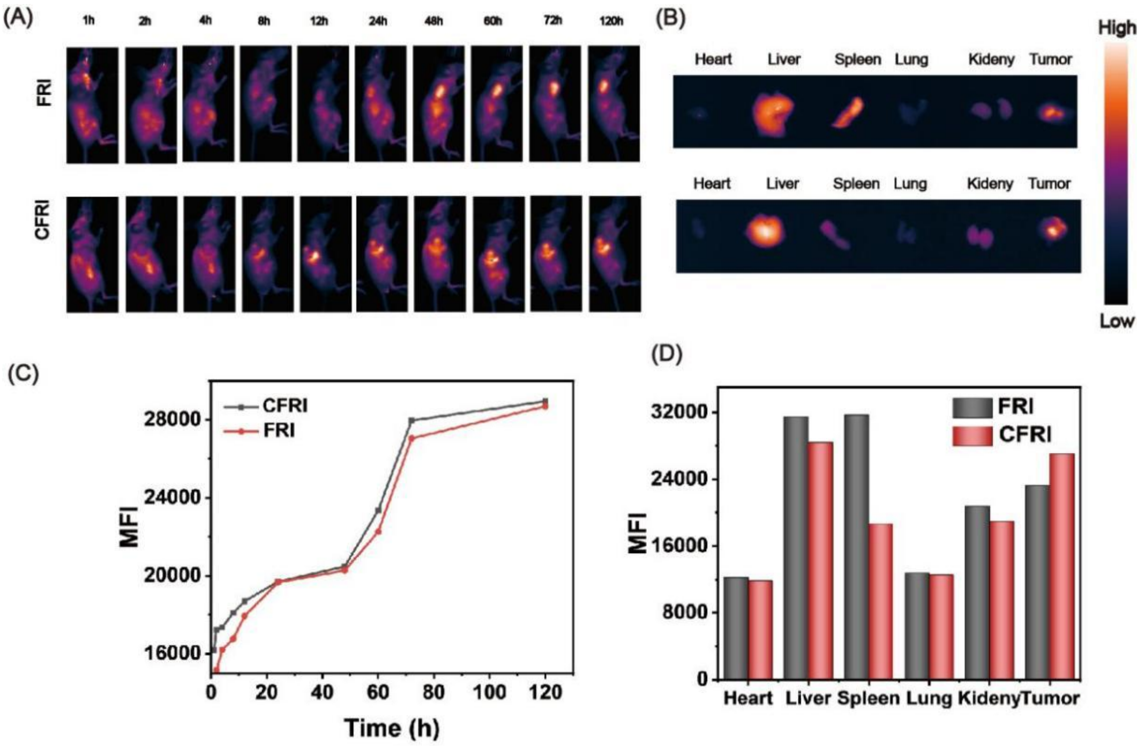

Fig. S8 *In vivo*, CFRI mediated biological distribution and photothermal imaging. (A) NIR fluorescence imaging of subcutaneous 4T1 tumor-bearing mice at different time points after in situ injection of FIR1048 or CFRI. (B)

In vitro fluorescence imaging of tumors and major organs (heart, liver, spleen, lung, kidney) in 4T1 tumor-bearing mice after intravenous injection for 120 h. (C) Quantitative analysis of the fluorescence intensity in 4T1 tumors. (D) The fluorescence intensity of tumors and major organs.

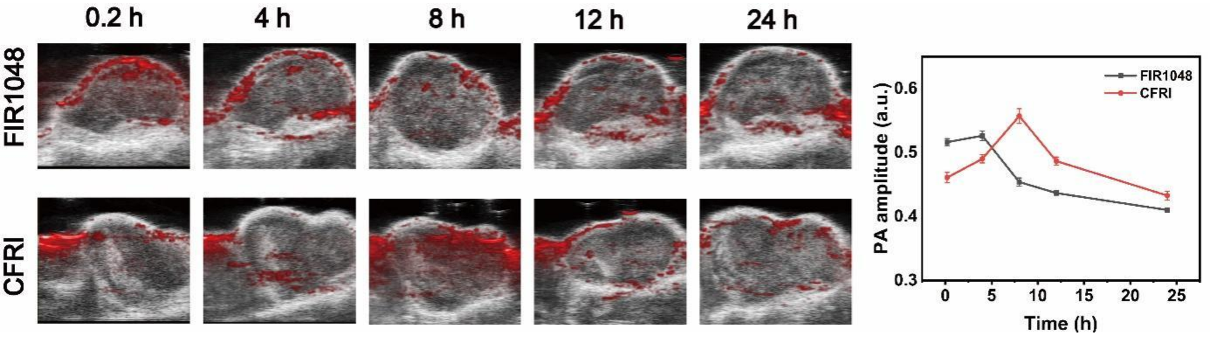

Fig. S9 PA image and signal intensity changes within 24 h after injection of FIR1048 or CFRI into the tumor site of 4T1 tumor-bearing mice.

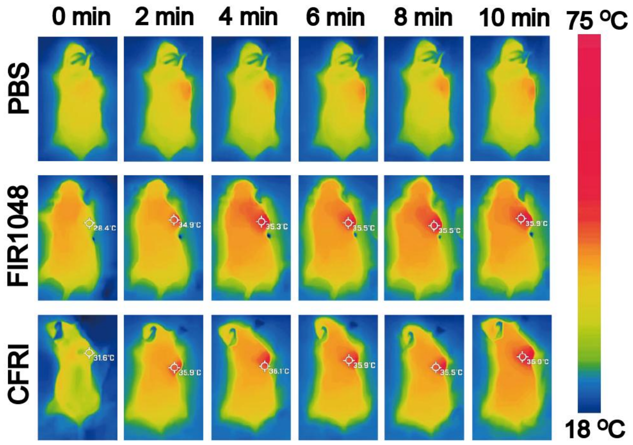

Fig. S10 4T1 tumor bearing mice were irradiated by injection of PBS, FIR1048 and CFRI under 1064 nm laser (1.0 W/cm^2, 10 min).

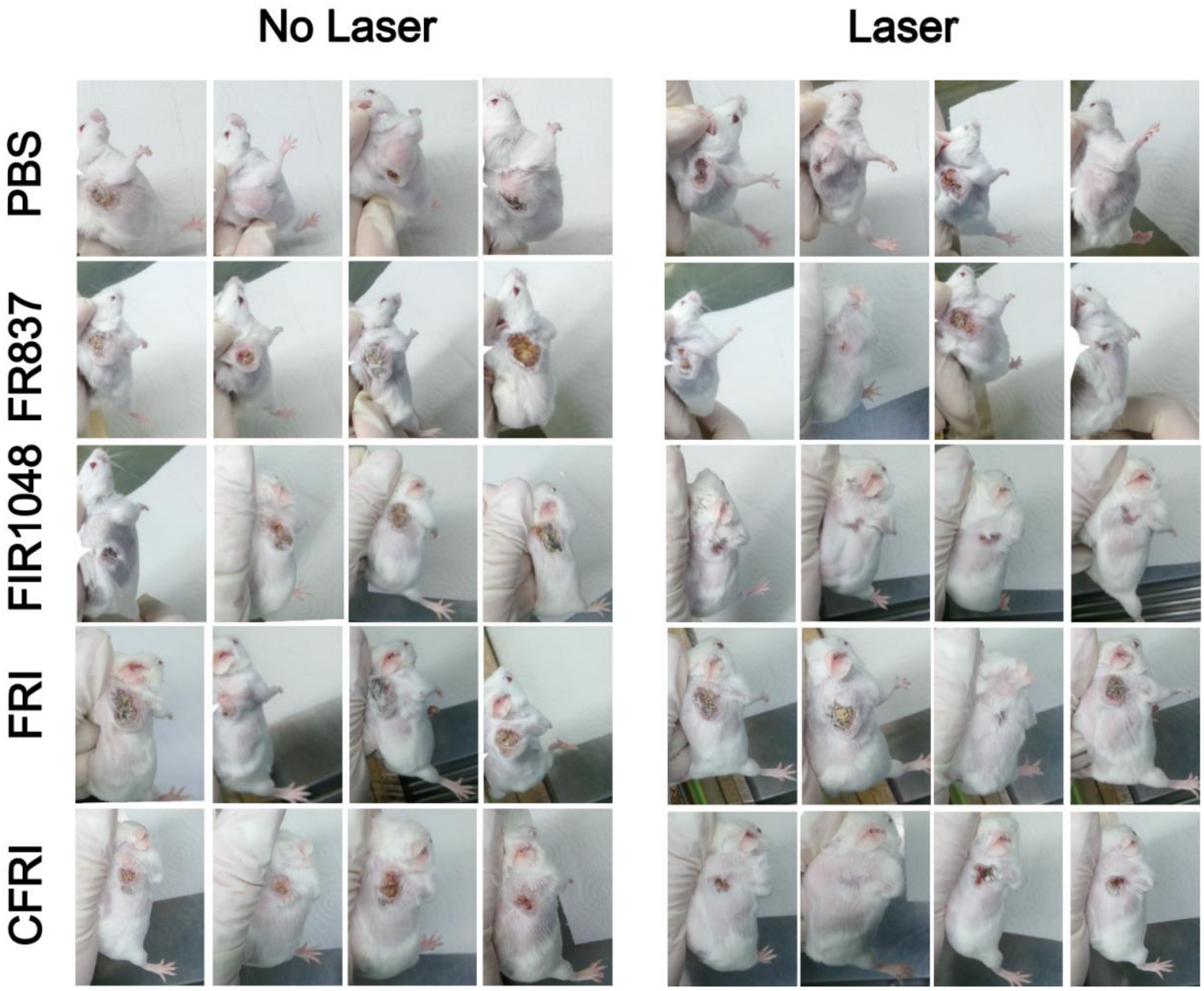

Fig. S11 The photographs of the tumors from mice after different treatments (PBS, PBS + L, FR837, FR837 + L, FIR1048, FIR1048 + L, FRI, FRI + L,CFRI, and CFRI + L).

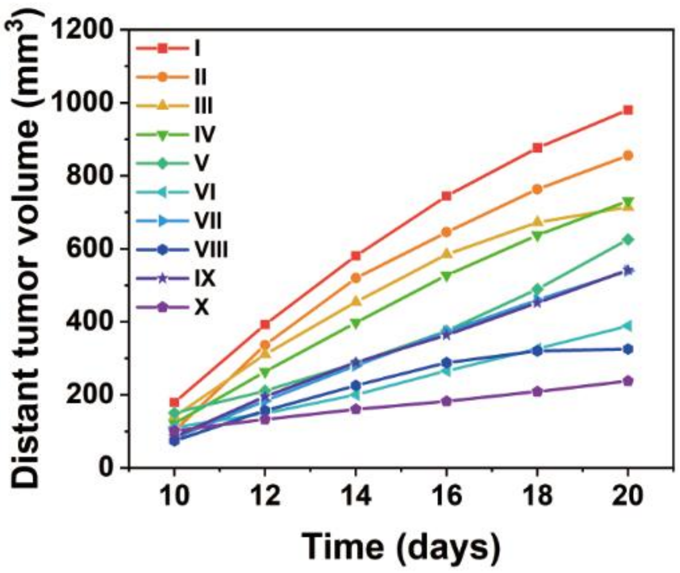

Fig. S12 The volume changes of distal tumors in 4T1 tumor-bearing mice treated with different materials. Group information: I. PBS; II. PBS + L; III. FR837; IV. FR837 + L; V. FIR1048; VI. FIR1048 + L; VII. FRI; VIII. FRI + L; IX. CFRI; X. CFRI + L.

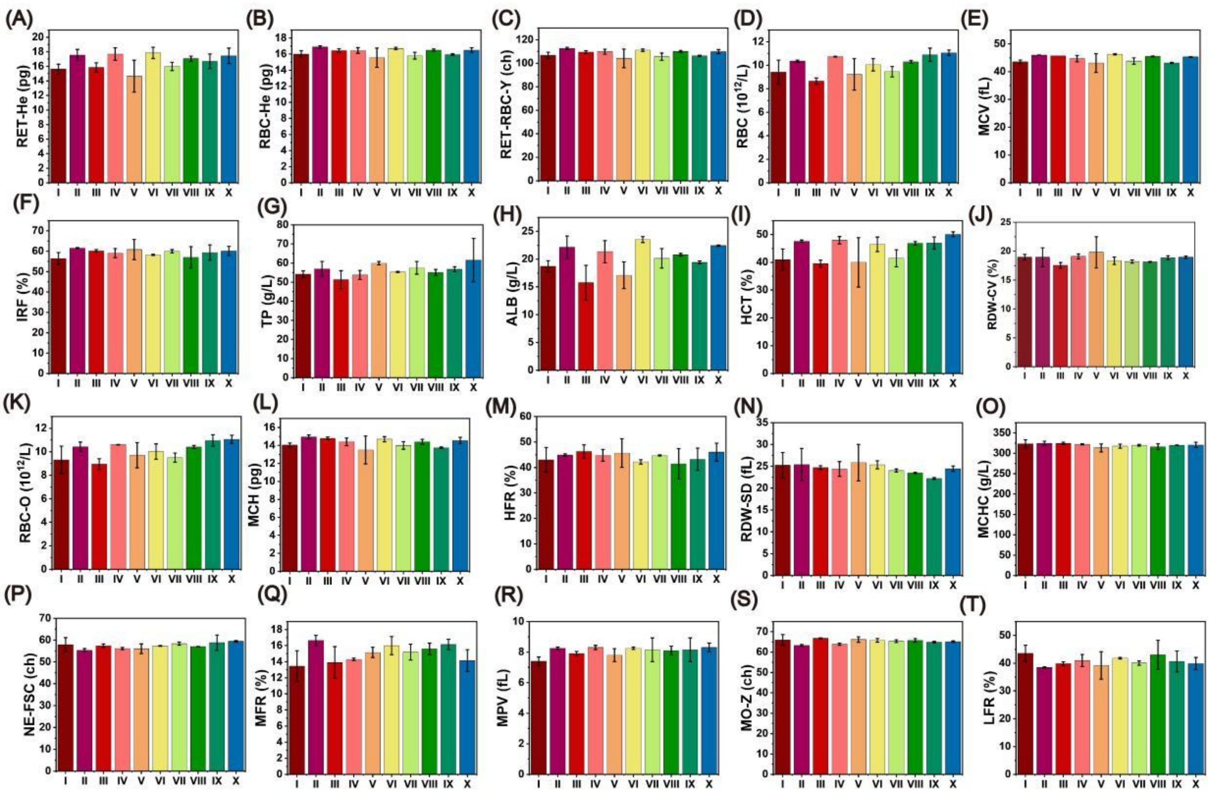

Fig. S13 Blood routine and biochemical analyses with different treatments. Group information: I. PBS; II. PBS + L; III. FR837; IV. FR837 + L; V. FIR1048; VI. FIR1048 + L; VII. FRI; VIII. FRI + L; IX. CFRI; X. CFRI + L.

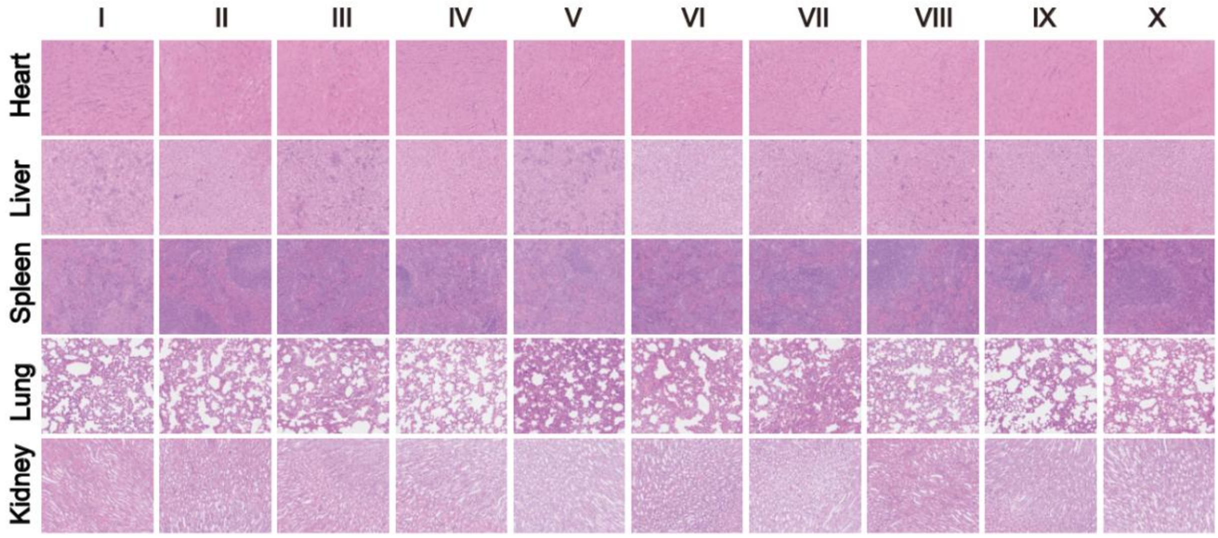

Fig. S14 H&E staining images of heart, liver, spleen, lungs, and kidneys of 4T1 tumor-bearing mice treated with different materials. Group information: I. PBS; II. PBS + L; III. FR837; IV. FR837 + L; V. FIR1048; VI. FIR1048 + L; VII. FRI; VIII. FRI + L; IX. CFRI; X. CFRI + L.

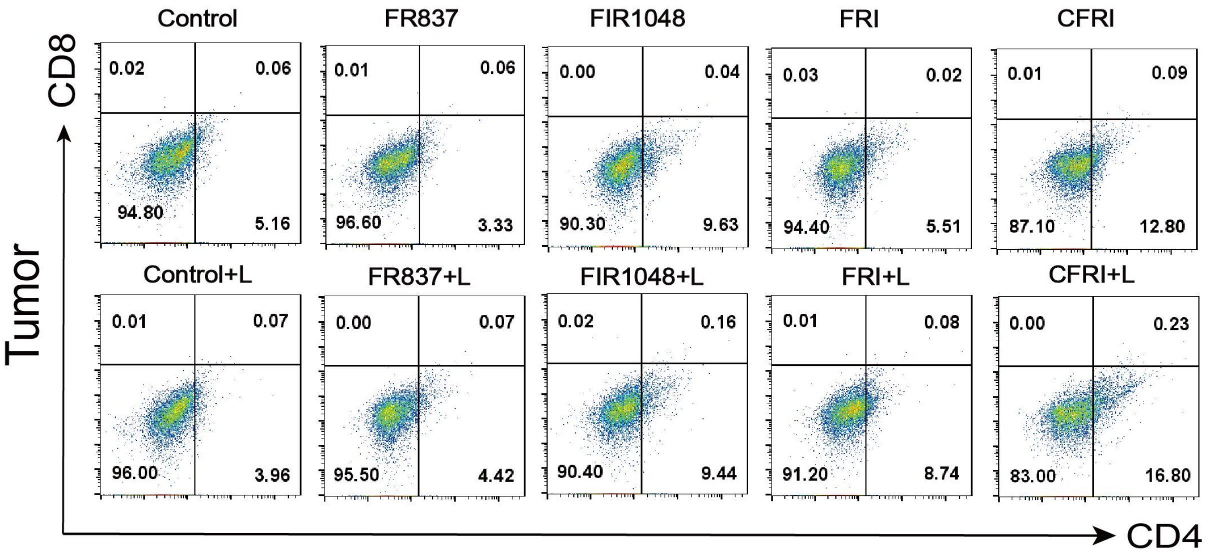

Fig. S15 Flow cytometry analysis of the proportion of CD4^{+} and CD8^{+} T cells in the primary tumors with different treatments.

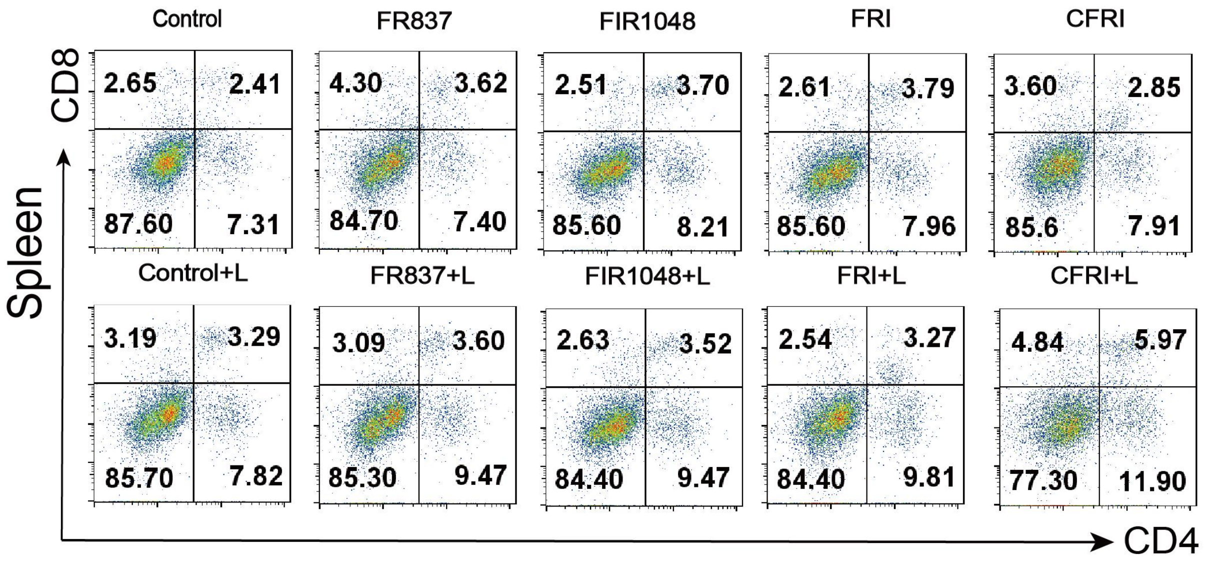

Fig. S16 Flow cytometry analysis of the proportion of CD4^{+} and CD8^{+} T cells in spleens with different treatments.
